# Children Probably Store Short Rather Than Frequent or Predictable Chunks: Quantitative Evidence From a Corpus Study

**DOI:** 10.3389/fpsyg.2019.00080

**Published:** 2019-01-30

**Authors:** Robert Grimm, Giovanni Cassani, Steven Gillis, Walter Daelemans

**Affiliations:** Department of Linguistics, Computational Linguistics and Psycholinguistics Research Center, University of Antwerp, Antwerp, Belgium

**Keywords:** segmentation, undersegmentation, chunks, multi-word units, formulaic language, age of first production

## Abstract

One of the tasks faced by young children is the segmentation of a continuous stream of speech into discrete linguistic units. Early in development, syllables emerge as perceptual primitives, and the wholesale storage of syllable chunks is one possible strategy for bootstrapping the segmentation process. Here, we investigate what types of chunks children store. Our method involves selecting syllabified utterances from corpora of child-directed speech, which we vary according to (a) their length in syllables, (b) the mutual predictability of their syllables, and (c) their frequency. We then use the number of utterances within which words are contained to predict the time course of word learning, arguing that utterances which perform well at this task are also more likely to be stored, by young children, as undersegmented chunks. Our results show that short utterances are best-suited for predicting when children acquire the words contained within them, although the effect is rather small. Beyond this, we also find that short utterances are the most likely to correspond to words. Together, the two findings suggest that children may not store many complete utterances as undersegmented chunks, with most of the units that children store as hypothesized words corresponding to *actual* words. However, dovetailing with an item-based account of language-acquisition, when children do store undersegmented chunks, these are likely to be short sequences—not frequent or internally predictable multi-word chunks. We end by discussing implications for work on formulaic multi-word sequences.

## 1. Introduction

The present study investigates undersegmented chunks in child language development. Previous work suggests that young children sometimes store speech sequences such as *Oh dear* or *Where's it gone* as internally unanalyzed chunks, without having discovered smaller constituents such as words or phonemes (Lieven et al., 1992; Pine and Lieven, 1993).

This is, most likely, a result of word segmentation: When children do not yet know what the meaningful units in their language are, they could initially store some speech sequences as undersegmented chunks, which are then further analyzed by comparing chunks to known lexical items (MacWhinney, 1978; Peters, 1983). In this paper, we are interested in the nature of such undersegmented chunks.

As one possibility, children could extract and store frequently recurring speech sequences. Frequency effects are pervasive in language development, with children acquiring frequent words, morphemes, and even syntactic constructions before less frequent exemplars (Ambridge et al., 2015). Consequently, it would be sensible to expect preferential storage of particularly frequent chunks. Alternatively, perhaps frequency is less important than input properties that indicate whether a given sequence corresponds to a discrete linguistic unit, such as a word or a morpheme. Next to frequent chunks, we thus consider two other, potentially more word-like chunk types: especially internally predictable and particularly short chunks.

A landmark study by Saffran et al. (1996) first demonstrated that children can exploit conditional probabilities between adjacent syllables in order to extract nonsense words from a continuous stream of speech. This raises the more general possibility that, during the word segmentation process, children use syllabic predictability to extract multi-syllable chunks from speech. Chunks with high syllabic predictability might be more likely to correspond to discrete linguistic units than frequent syllable sequences, and perhaps this inclines children to store internally predictable rather than frequent undersegmented chunks.

Yet another possibility is that stored chunks are neither frequent nor predictable, but simply short. Children's memory for speech sequences is likely to be limited with respect to the length of memorized material, and this could predispose them toward preferentially storing short chunks. This argument forms part of MacWhinney (1978, 2014)'s theory of item-based learning, wherein children initially extract short phrases as single lexical items (i.e., as chunks) and further analyze extracted chunks via comparison to known items.

Relative to longer speech sequences, short sequences are also unlikely to contain smaller linguistic units and might thus appear more word- or morpheme-like to the language-acquiring child. As a consequence, perhaps children are more likely to store short rather than frequent sequences as undersegmented chunks. In this study, we investigate all three chunk properties—(1) whole-chunk frequency, (2) syllabic predictability, (3) chunk length—, and we ask to what extent children rely on these properties during the extraction of an initial chunk vocabulary.

The remainder of the paper is structured as follows. First, we survey evidence for the existence of unsegmented chunks in young children, arguing that they emerge as a by-product of word segmentation. Following this, we provide a brief sketch of our method, which involves selecting multi-syllable utterances as sequences that could potentially be stored (by young children) as undersegmented chunks. Varying the syllabic predictability, frequency, and length of selected utterances, we evaluate which multi-syllable utterances (henceforth *MSUs*) perform better at predicting the time course of word learning.

Our method extends previous work by Grimm et al. (2017), who found that words contained in a large number of multi-word phrases tend to be learned early in development. Referring to Peters (1983), Grimm et al. (2017) suggest that children store some phrases as undersegmented chunks. Chunks are then compared to one another in order to identify shared sub-units. And the more chunks contain a particular unit (such as a word), the easier it should be to discover that unit. We expand on this by evaluating whether short, frequent, or internally predictable MSUs perform better at predicting when their constituent words are learned—arguing that well-performing MSUs are more likely to be stored within children's early proto-lexica.

### 1.1. Evidence for Children's Unanalyzed Chunk Vocabularies

Young children sometimes produce utterances in ways which suggest that they are treated as (partially) unanalyzed wholes. Peters (1983) surveyed various examples, including e.g., the child utterance *I don't know where's Emma one*, which appears to consist of the previously heard utterances *I don't know* and *Where's Emma one*; or *I all very mucky too*, given in response to the statement *We're all very mucky*^1^. Observations like these suggest that children could extract and store in memory (a subset of) uninterrupted speech sequences. Children might then bootstrap a vocabulary of smaller units by comparing stored chunks to one another and to incoming speech—a proposal, going back to MacWhinney (1978), which Peters (1983) discusses as a possible strategy for early speech segmentation.

The idea receives support from a systematic investigation conducted by Lieven et al. (1992), who analyzed the productive vocabularies of twelve English-speaking children through parental reports and analyses of child-caregiver interactions. Child-produced multi-word utterances were coded as *frozen phrases* if they contained at least two words which had not previously occurred in isolation within the vocabulary of the child—or if they contained only one such word, so long as the word had not occurred in the same position within a previous utterance. Lieven et al. (1992) found that their subjects' productive vocabularies, at 50 and 100 produced units (phrases or words), contained around 20% frozen phrases. This reliance on frozen chunks, although practiced to different degrees by different subjects, seems to be a strategy shared by all children (Pine and Lieven, 1993).

By comparing stored chunks to other items, MacWhinney (1978) proposed, children could discover chunk-internal positions of variability (*slots*). Within the theory of item-based learning (MacWhinney, 1978, 2014), slots are attached to predicates and can be filled by arguments (e.g., *object*, as in the pattern *give me + object*). Lieven et al. (2009) implemented a similar idea in a computational method that reconstructs child utterances on the basis of earlier productions. The method first attempts to match a given utterance with earlier child productions and, if this is not possible, inserts abstract slots. For example, upon observing the utterances *I go bathroom* and *I go home*, it could create an *I go + location* construction. Lieven et al. (2009) report that between 20 and 40% of their 2-year-old subjects' utterances could be exactly matched to previous productions, while the majority of non-exact matches required the insertion of just a single slot. These results are echoed by Bannard et al. (2009) and Borensztajn et al. (2009), who also worked with child-produced speech and applied methods for grammar induction that can discover both lexicalized and abstract constituents.

The early building blocks of child language, then, appear to include unanalyzed chunks. Such findings can be situated within a usage-based approach to language acquisition (Behrens, 2009; Tomasello, 2009), a framework which conceives of early linguistic representations as lexically specific units that often span multiple words. Representations are refined and become more abstract over time, and the developed cognitive system operates with both lexically specific and more abstract patterns^2^. Unanalyzed chunks, that is, should only exist for a short developmental window, when children are faced with the task of segmenting continuous speech into discrete units. But once that process is complete, smaller linguistic units should replace the initial chunk vocabulary. We next review converging evidence from empirical studies and computational models of word segmentation in support of this notion.

### 1.2. Undersegmented Chunks During Word Segmentation

One of the first challenges faced by children during language development is what Peters (1983) called the *initial extraction problem*: Without knowledge of the units in their target language(s), which speech sequences should children pick out as hypothesized linguistic units? Early perception studies showed that 2-month-olds demonstrate improved discrimination of syllable-like sequences (Bertoncini and Mehler, 1981) and are proficient at storing information pertaining to the syllabic—but not the phonemic—structure of speech (Jusczyk and Derrah, 1987). Follow-up work suggests that even 4-day-old neonates perceive speech in terms of syllables (Bijeljac-Babic et al., 1993). And on the computational modeling side, it is possible to segment speech into units that closely resemble syllables by tracking changes in sonority (Räsänen et al., 2015, 2018)—i.e., by attending only to changes in audibility, without reliance on prior linguistic knowledge. The syllable thus presents a good candidate for an early perceptual primitive in speech.

As one possible segmentation strategy, children could focus on sequences characterized by high transitional probabilities (TPs) between syllables^3^. In a seminal study, Saffran et al. (1996) exposed 8-month-olds to synthesized streams of nonsense words, with no cues to word boundaries other than the co-occurrence patterns of syllables. Within-word TPs of four different three-syllable nonsense words (e.g., *padoti* or *golabu*) were 1.0 (e.g., *go* was always followed by *la*), while TPs between syllables spanning word boundaries were 0.33 (e.g., *bu* could be followed by the first syllable of three other words). In the testing phase, subjects listened longer to sequences which spanned word boundaries than to the more internally predictable nonsense words. Infants typically pay more attention to novel stimuli, and less to familiar ones. Saffran et al. (1996)'s results thus imply that subjects were familiar with the internally predictable nonsense words. Infants, that is, appear capable of exploiting statistical regularities between syllables to segment words from fluent speech. Aslin et al. (1998) replicated these results while keeping the frequencies of nonsense words constant, demonstrating that TPs provide a useful cue even when they are not correlated with frequency^4^.

There are, of course, other potential segmentation cues, such as stress or co-articulation (Johnson and Jusczyk, 2001; Thiessen and Saffran, 2003). Sensitivity to certain cues seems to be present at an early age, while other cues are only used at later stages. For example, 7-month-olds exhibit sensitivity to TPs but not to stress, while 9-month-olds can exploit stress patterns in an artificial segmentation task (Thiessen and Saffran, 2003). Thiessen and Saffran (2003) hypothesize that this indicates an early exploitation of statistical structure in order to extract a first set of words. These are then used to discover language-specific stress patterns, which can help to further segment the input. Extracted units *could* correspond to actual words, but this need not always be the case. Some units, extracted via reliance on statistical structure, could be stored as undersegmented chunks; and by comparing chunks to one another, children could discover language-specific segmentation cues, bootstrapping further segmentation. This bootstrapping approach to segmentation has the potential to explain other patterns in language development, such as the emergence of phonemic categories before the presence of a large receptive lexicon: If children approach segmentation by constructing a proto-lexicon of chunks, early phonemic contrasts could emerge as a result of identifying minimally different chunks (Martin et al., 2013).

Under such a proposal, undersegmented chunks are a side-effect of the segmentation process, and they would become fully analyzed once that process is complete. Evidence from computational models of word segmentation supports this view. The models described by Goldwater et al. (2009), for example, start from phoneme sequences, which are then segmented on the basis of statistical regularities between phonemes^5^. Discovered units include words, but also many undersegmented chunks. Another segmentation strategy, not mutually exclusive with reliance on statistical structure, is the wholesale storage and gradual breaking-down of full utterances. In this possible scenario, children initially store full utterances as holistic units, and novel input sequences are only split if another unit (stored in memory) is contained within them, leading to the discovery of more and more fine-grained units. Computational models which implement this strategy (Lignos and Yang, 2010; Monaghan and Christiansen, 2010; Lignos, 2012) achieve excellent performance^6^ and thus demonstrate how a large number of undersegmented chunks could accumulate as by-products of the segmentation process. There is even some tentative experimental evidence for chunk-based segmentation strategies in child language acquisition: 2-month-olds show improved memory for speech when it is contained in clause-like units, compared to being presented in list form or spanning clause boundaries (Mandel et al., 1994); and at the same time, it has been demonstrated that 6-month-olds can use their own name or the word *mommy* to segment unfamiliar words from novel sequences (Bortfeld et al., 2005).

Undersegmented chunks, in summary, are a plausible by-product of the segmentation process. In the current study, we ask which types of chunks children initially extract from speech. In language development, frequent items are generally learned before less frequent items (Ambridge et al., 2015), and one could thus expect children to preferentially extract and store frequent chunks. Support for the role of frequency during segmentation comes from findings that 8-month-olds can detect words within fluent speech on the basis of their frequency (Jusczyk and Aslin, 1995), and 11-month-olds are sensitive to highly frequent syllable sequences that span word boundaries as well as highly frequent disyllabic nonsense words (Ngon et al., 2013). While this does not necessarily mean that children store all frequent speech sequences as chunks, it nevertheless implies that frequency could be a major determinant in whether or not a given sequence is stored.

Perhaps, however, frequency is less important than the perceived unity of a given syllable sequence. That is, perhaps children store syllable sequences which appear to form a discrete unit and cannot, for all intents and purposes, be segmented into smaller units, such as words or morphemes. For example, we can reasonably expect that short sequences are more likely to correspond to words or morphemes than longer sequences. Thus, if children store chunks as hypothesized words or morphemes, perhaps they simply store uninterrupted speech sequences that happen to be particularly short.

Another argument for why children should favor shorter sequences is based on memory limitations. MacWhinney (1978, 2014) proposes, in the context of item-based learning theory, that restrictions on children's memory capacity should prevent them from fully storing (most) uncomprehended sequences; and that they might only store particularly short chunks—e.g., 2- or 3-word sequences—as single lexical items. This proposal alleviates concerns having to do with memory limitations and would seem to constitute a fruitful learning strategy for children to pursue, given that parental utterances tend to be relatively short (Saxton, 2010). Indeed, MacWhinney (2014) reports that close to a quarter of parental utterances in a corpus of English child-directed speech (containing approx. 500,000 words) are single-word utterances.

Thus, one of the claims of item-based learning is that children begin to acquire linguistic knowledge by extracting short speech sequences as unsegmented chunks. They can then discover novel words by splitting known items from stored chunks (MacWhinney, 1978, 2014). When a newly segmented word corresponds to a predicate—e.g., *your*, as in *Where are your pajamas?*—, the child may notice that the meaning she has assigned to the predicate only makes sense if it is combined with an argument. Here, the possessive meaning of *your* requires an argument that corresponds to the object being possessed. Based on the particular utterance within which *your* was encountered, this would prompt the child to acquire the item-based pattern *your + pajamas*; and as she encounters the predicate in conjunction with other words, the pattern would broaden to accommodate a range of possible words (*your + object*).

Item-based learning, then, posits that children's early lexical and syntactic development is derived from short input sequences, as novel words and new syntactic patterns are both acquired from short chunks. From this point of view, we should expect children to be biased toward storing particularly short chunks—and not necessarily frequent or internally predictable sequences.

Alternatively, given that TPs are an early segmentation cue (Saffran et al., 1996; Aslin et al., 1998; Thiessen and Saffran, 2003), children might extract and store sequences whose syllables are especially mutually predictive—even if the entire syllable sequence is relatively long or infrequent. Syllable sequences presumably exist along a spectrum of predictability, with some consisting of syllables that always and only occur with one another, while others have a more variable internal structure. If the goal of segmentation is the discovery indivisible units, then sequences with stronger internal predictability might be more likely to be considered as hypothesized words or morphemes—and therefore to be stored as chunks.

## 2. Goal and Method

We consider the following research question: When extracting undersegmented chunks from speech during first language acquisition, are children more likely to extract (a) frequent, (b) internally predictable, or (c) short syllable sequences? We investigate (a) because frequent items, being acquired before less frequent exemplars (Ambridge et al., 2015), may simply be associated with a general learning advantage. We chiefly examine (b) and (c), on the other hand, because children might be biased to extract discrete linguistic units from unsegmented input; and short or predictable sequences, in contrast to frequent items, should have a higher chance of corresponding to such units.

Before answering the core research question, we first attempt to verify the assumption that short and predictable MSUs are more word-like than frequent MSUs. This is done by selecting various sets of multi-syllable utterances (MSUs) from the input English-speaking children typically receive. We refer to these as *chunk sets*—selections of uninterrupted syllable sequences which children could potentially store as chunks. If we are correct in assuming that short and internally predictable MSUs are more word-like, we should find that chunk sets with short and predictable MSUs are better-suited for selecting single-word utterances than sets with frequent MSUs.

After examining which types of chunk sets contain more words, we evaluate the likelihood that children store the MSUs in a given chunk set as unanalyzed units. One difficulty with devising such a method is that chunks might only be stored for brief periods and might only rarely be produced, if children use them at all. Because of this, methods for tapping into the chunk vocabulary of children should not rely on child productions. Instead, we evaluate MSUs according to how well they perform at predicting when their constituent words are learned.

This method has previously been introduced by Grimm et al. (2017), who used an existing computational model (McCauley and Christiansen, 2014) to extract multi-word phrases from corpus data. Extracted phrases were used to predict the developmental stage at which children learn to produce the words contained within them. For this purpose, the incidence of the phrases containing each word was determined and correlated with the developmental stages at which children first produce the words. The correlation is negative, even when controlling for the frequency of words—i.e., words contained in many different phrases tend to be learned earlier than words contained in fewer phrases.

By way of explanation, Grimm et al. (2017) refer to segmentation: If phrases are stored as chunks, it should be easier to identify words contained in a large number of phrases, relative to words contained in fewer phrases. This would follow from an approach to word segmentation wherein the comparison of stored chunks leads to the detection of common sub-sequences—an idea that was introduced in item-based learning, where children split known items from unsegmented sequences (MacWhinney, 1978, 2014); and that is subsequently discussed in Peters (1983)'s work, who refers to it as *phonological matching*.

Assuming *phonological matching*, encountering a particular sub-sequence within many chunks could be advantageous in at least two ways: (1) Finding a particular sub-sequence within many different chunks might strengthen its hypothesized status as an independent unit; and (2) the more chunks contain a given word, the greater the chance that units which are encountered in the future can be split from one of those chunks—a strategy infants could, in principle, use during segmentation (Bortfeld et al., 2005).

Expanding on this, we evaluate chunk sets according to how well included MSUs perform at predicting the age at which children first produce the words contained within them. MSUs which are stored as chunks should perform well, whereas those that are never stored should perform poorly. Thus, whole-sequence frequency will be implied as a determinant of chunkhood to the extent that chunk sets containing frequent MSUs can predict when their component words are learned; syllabic predictability will be implied to the extent that internally predictable MSUs predict word learning; and sequence length will be implied to the extent that chunk sets with short MSUs predict word learning.

## 3. Analysis I: Chunk Selection

In this analysis, we describe the method used to select chunk sets, which we define as subsets of the MSUs found in English child-directed speech. Our method involves ranking MSUs by (1) syllable length, (2) syllable predictability, and (3) frequency—followed by selecting the top *N* MSUs from each ranking.

### 3.1. Method

To select chunk sets from English child-directed speech (CDS), we rely on three properties: (1) the overall frequency of MSUs in CDS, (2) their length in syllables, and (3) the average predictability of adjacent syllables. Given a set of MSUs from a corpus of CDS, we rank MSUs by (1)–(3), and we select the top *N* items from each ranking. MSUs are ranked from most to least frequent, from shortest to longest, and from most to least predictable. We thus obtain three chunk sets—corresponding to the *N* most frequent, *N* shortest, and *N* most internally predictable MSUs.

#### 3.1.1. Corpora

We extract MSUs from transcribed CDS, which differs markedly from the speech used by adults to address other adults. Among other things, CDS consists of shorter phrases, contains more pauses, and is composed of a more limited vocabulary (Saxton, 2010). Its properties appear to facilitate word segmentation and word learning (Thiessen et al., 2005; Yurovsky et al., 2012), making it the obvious corpus choice. We obtain CDS samples from various corpora of transcribed speech exchanged between caretakers and young children, taken from the CHILDES database (MacWhinney, 2000a). A typical corpus consists of various transcripts based on interactions (e.g., reading a book, playing a game) involving a child or group of children and their caretakers. Given that individual corpora contain at most a few hundred thousand words, we collapse various English CHILDES sources into a North American corpus (NA corpus) and a British English corpus (BE corpus)^7^. Since most corpora in the CHILDES database are transcribed at the word level, whereas we are interested in processes which precede the segmentation of speech into words, we syllabify all corpora—motivated by the observation that neonates and infants perceive speech in terms of syllables (Bertoncini and Mehler, 1981; Jusczyk and Derrah, 1987; Bijeljac-Babic et al., 1993). We convert each word to a syllable representation by relying on a syllabified version of the Carnegie Mellon University (CMU) pronouncing dictionary (Bartlett et al., 2009)^8^. We keep only those utterances whose words have an entry in the CMU dictionary. About 80% of utterances survive this syllabification process. Table 1 summarizes other relevant statistics.

**Table 1:** Child-directed speech statistics.

**Measure**	**BE**	**NA**
# adult speakers	280	737
# children addressed	247	743
mean child age (months)	32.66 (*SD* = 9.25)	41.39 (*SD* = 23.45)
# utterances	1,467,445	1,319,102
mean utterance length (words)	4.55 (*SD* = 3.69)	4.46 (*SD* = 3.46)
# tokens	6,690,453	5,890,443
# types	49,206	35,699
# syllabified utterances	1,190,858	1,083,618
mean utterance length (words)	4.42 (*SD* = 3.45)	4.08 (*SD* = 3.09)
# syllabified tokens	5,266,479	4,428,993
# syllabified types	19,931	14,156

#### 3.1.2. Possible Chunks

We consider full utterances from CDS as possible chunks, i.e., as syllable sequences from which to select chunk sets. Sampling smaller sequences would require mechanisms for decomposing utterances and could confound the results. For example, a decomposition based on TPs would pre-suppose that children prioritize syllable predictability when extracting chunks from speech. Working with full utterances avoids this problem. Moreover, the storage of utterances presents an easy solution to Peters (1983)'s *initial extraction problem*: If children have no knowledge about linguistic units, the most straightforward hypothesis is to consider uninterrupted stretches of speech as potential units. We thus consider MSUs from CDS as candidates for inclusion in chunk sets.

However, to lessen the probability that included utterances are not idiosyncratic to particular child-caretaker dyads, we require that MSUs are produced by adults from at least two different CHILDES corpora. This reduces the number of available utterances, in the BE and NA corpus, from more than 1,000,000 to about 50,000 each. The reason for this fairly drastic step lies in the nature of our corpus material: Because we collapse data from a large number of different CHILDES corpora (10 for the BE and 41 for the NA corpus), with hundreds of child and adults speakers, most MSUs will not form part of the input received by the children addressed in the different corpora. For example, the BE corpus contains the adult-produced MSU, *On Wednesday he ate through three plums*. Unsurprisingly, this MSU is only used once, to address a particular child, in a situation that is unlikely to occur with any of the other children whose input we are considering. Because of this, it would not make sense to include it as an utterance that children could, in general, store as an undersegmented chunk. Thus, to reduce the likelihood that such idiosyncratic MSUs are included in the aggregated BE and NA corpora, we filter MSUs by the number of individual CHILDES corpora within which they occur—requiring them to be used, on independent occasions, by caretakers from at least two of the (41 + 10 = 51) CHILDES corpora.

Furthermore, given that we consider the syllable as a primitive unit, single-syllable utterances are already fully segmented and cannot be considered as undersegmented chunks. For this reason, we require that the utterances included in chunk sets contain at least two syllables (i.e., we consider *multi-syllable* utterances/MSUs). Finally, to control for repetition, we exclude MSUs that consist of repeated occurrences of a single word. The three criteria (more than one syllable, no repetitions, used in at least two CHILDES corpora) are met by 50,199 MSUs in the BE corpus and by 57,151 MSUs in the NA corpus.

#### 3.1.3. Selection of Chunk Sets

From the available MSUs, we wish to select the *N* most frequent, *N* shortest, and *N* most internally predictable items as chunk sets. We thus need to fix the size of each chunk set to some *N*, where *N* must be smaller than the number of all MSUs. Otherwise, there would only be one chunk set, and it would contain all MSUs. Given some *N*, we then select MSUs according to their frequency, their length in syllables, and the mutual predictability of their syllables. We determine frequency by counting how often MSUs appear in CDS, length by counting the number of syllables in each MSU, and predictability by averaging over the conditional probabilities between adjacent syllables.

More formally, each syllable *u*_*i*_ within the MSU *u*_1_, *u*_2_…*u*_*n*_ can be associated with a set *P*_*i*_ of conditional probabilities:

$$P_{i}=\{\begin{array}{ll} \left\{p(u_{i}|u_{i-1}),p(u|u_{i+1})\right\} & \text{if }i>1\wedge i<n\\ \left\{p(u_{i}|u_{i-1})\right\} & \text{if }i>1\wedge i=n\\ \left\{p(u_{i}|u_{i+1})\right\} & \text{if }i=1\wedge i<n \end{array}$$

The predictability score of a given MSU is then defined as the average of the conditional probabilities associated with the syllables in a given MSU. This definition is inspired by the oft-replicated finding that infants are sensitive to the TPs (conditional probabilities) between syllables (Saffran et al., 1996; Aslin et al., 1998; Thiessen and Saffran, 2003), suggesting that the local predictability of syllables within sequences is an early segmentation cue.

At this point, *N* is an obvious tweakable parameter. As mentioned, *N* must be smaller than the number of available MSUs. Otherwise, the only chunk set would contain all MSUs, and we would not be able to distinguish between especially frequent, short, or internally predictable MSUs. At the same time, *N* should not be extremely small either. For example, it would not make sense to set *N* = 1. But even values in the tens or hundreds might not be sufficiently large. Since we wish to predict the age at which words are learned from the number of MSUs within which these words are contained, it would be good to operate with fairly large chunk sets, to ensure that a majority of target words will in fact appear within some MSU. For the current illustrative purpose, we set N = 10,000. In subsequent analyses, however, we report results for many possible choices of *N*.

### 3.2. Results and Discussion

To illustrate the chunk set selection procedure, we focus on example sets from the BE corpus—consisting of the N = 10,000 shortest MSUs, the *N* most frequent MSUs, and the *N* most predictable MSUs. Table 2 summarizes statistics pertaining to the three sets. As expected, the average syllable count of the *N* shortest MSUs is lowest; the average frequency count of the *N* most frequent MSUs is highest; and the average predictability score the of *N* most internally predictable MSUs is largest. Overlap between the three sets is limited to below 30%, indicating that the chunk sets contain fundamentally different types of MSUs.

**Table 2:** Statistics for chunk sets with the N = 10,000 shortest, most frequent, and most predictable MSUs.

**Chunk set**	**Short**	**Frequent**	**Predictable**
Mean frequency	18 (SD: 212)	**31** (SD: 220)	13 (SD: 179)
Mean predictability	0.10 (SD: 0.14)	0.15 (SD: 0.13)	**0.30** (SD: 0.09)
Mean length (syllables)	**2.36** (SD: 0.48)	3.73 (SD: 1.45)	4.42 (SD: 1.61)
Overlap with shortest	–	28.6%	15.49%
Overlap with most frequent	–	–	25.5%
Overlap with most predictable	–	–	–

*The largest value in each column are given in bold-face*.

Table 3 contains example MSUs from each chunk set^9^. The most predictable MSUs (e.g., *brilliant, breakfast*) correspond to syllable sequences whose component syllables, if they do occur, have a high chance of occurring within the given MSUs. For example, the syllable corresponding to *brill-* occurs only to the left of the syllable corresponding to *-iant*, and the syllable corresponding to *-iant* occurs only to the right of the syllable corresponding to *brill-*. This means that the conditional probabilities associated with the two syllables are both 1.0, leading to a 1.0 average predictability score for *brilliant*. Strikingly, the 15 most internally predictable MSUs all correspond to individual words—with both very high and very low frequency counts.

**Table 3:** Top 15 MSUs from chunk sets containing the (1) *N* shortest, (2) *N* most frequent, and (3) *N* most internally predictable MSUs.

****N**** **shortest**			****N**** **most frequent**			****N**** **most predictable**		
**MSU**	**freq**	**pred**	**MSU**	**freq**	**pred**	**MSU**	**freq**	**pred**
More bricks	9	0.03	Okay	12,101	0.57	Vampire	3	1.00
Push out	1	0.00	Uhu	7,613	0.98	Brilliant	317	1.00
Nice tea	2	0.00	That's right	7,474	0.20	Breakfast	30	1.00
Quiet	23	0.86	Pardon	5,033	0.75	Trowel	4	0.99
Stop there	3	0.00	That's it	4,823	0.08	Uhu	7,613	0.98
A leg	2	0.02	Come on	4,734	0.31	Grandad	35	0.97
Bread yeah	1	0.00	Oh dear	4,697	0.46	Children	13	0.96
Train what	2	0.00	What's that	3,747	0.19	Fraser	1,627	0.96
Left eye	2	0.00	Thank you	3,002	0.51	Nonsense	4	0.96
Right back	3	0.00	Oh no	2,945	0.04	Hello	1,680	0.95
London	15	0.49	Good girl	2,293	0.36	Jigsaw	15	0.95
Red bear	2	0.00	There you go	2,262	0.10	Hungry	6	0.94
What room	1	0.00	I don't know	2,248	0.19	Costume	4	0.94
The farm	2	0.21	What is it	2,225	0.13	Husband	1	0.94
Window	20	0.81	Is it	2,151	0.20	Croissant	10	0.94

The 15 most frequent MSUs, on the other hand, include single words (e.g., *okay*) and idiomatic sounding multi-word utterances (e.g., *oh dear, I don't know*). The much lower predictability scores associated with these MSUs indicate that their syllables are less strongly tied to one another: Even though MSUs such as *I don't know* are frequently used, the syllables corresponding to *I, don't* and *know* are also frequently used in MSUs other than *I don't know*. The shortest MSUs, finally, correspond to both disyllabic words (e.g., *quiet, window*) as well as disyllabic multi-word utterances (e.g., *stop there*). Since these MSUs are only selected according to length in syllables, their frequency counts and predictability scores are quite variable.

## 4. Analysis II: Which Multi-syllable Utterances Correspond to Single Words?

In considering frequent, predictable, and short MSUs, we have been assuming that the latter two MSU types are more word-like than the former. The previous analysis certainly suggests that the most internally predictable MSUs are more word-like than the most frequent MSUs—with the top 15 predictable items all corresponding to single-word utterances (cf. Table 3). It is possible, however, that the top 15 MSUs are special cases, with fewer single-word utterances among the MSUs further down the rank distribution. In the current analysis, we use a more rigorous method to determine which of the three metrics (syllable length, frequency, or syllabic predictability) is best-suited for selecting MSUs that correspond to single words. If our initial assumption is correct, MSU length and syllabic predictability should be better-suited for selecting single-word MSUs than whole-sequence frequency.

### 4.1. Method

Any method used to establish which of the three metrics is most useful for selecting single-word MSUs should address two key issues: (1) the need for an appropriate performance metric, and (2) the potentially confounding effect of the tweakable parameter *N* (chunk set size). We address both issues below.

#### 4.1.1. Classification Metrics

We would like to quantify whether different types of chunk sets—i.e., subsets of the available MSUs—are well-suited for selecting single-word MSUs. In the best case, a given chunk set will contain all and only single-word MSUs; and in the worst case, it will not contain any single-word MSUs. We can thus frame the selection of chunk sets as a classification task, where MSUs included in a particular chunk set are classified as *words*, and excluded MSUs are classified as *non-words*.

To quantify classification performance, we use a *precision* and a *recall* metric: the proportion of words contained within a given chunk set, and the proportion of words correctly selected out of all available words. More formally, let *N* be the chunk set size, *W*_*C*_ the number of words within the chunk set, and *W* the number of words outside of the chunk set. Precision and recall are then defined as follows:

(1) $$\begin{array}{r} Precision=\frac{W_{C}}{N} \end{array}$$

(2) $$\begin{array}{r} Recall=\frac{W_{C}}{W_{C}\;+W} \end{array}$$

Precision equals 1.0 if and only if the chunk set contains only single-word MSUs, and recall equals 1.0 if and only if the chunk set contains all available single-word MSUs. A chunk set that contains all and only single-word MSUs will thus lead to maximum precision and recall. To quantify the notion that well-performing chunk sets should maximize both precision and recall, we track overall classification performance via the *F-score*, defined as the harmonic mean of precision and recall (a measure of classification performance commonly used in computational linguistics and studies investigating speech segmentation in children—see e.g., Goldwater et al. (2009) and the references therein). While we do not expect to achieve maximum scores with our chunk sets, we nevertheless expect to obtain informative differences between classification outcomes.

#### 4.1.2. Effect of Chunk Set Size

*N* (chunk set size) could in principle take any value between 1 and the total number of MSUs (50,199 for the BE corpus and 57,151 for the NA corpus). Crucially, robust results should emerge across all choices of *N*—excluding only large and small values. Large values close to the number of all available MSUs should lead to similar result for the three chunk sets, since each set will contain the same selection of MSUs. But *N* should not be too small either: The BE and NA corpus contain 1,856 and 2,159 single-word MSUs, respectively, and chunk sets containing fewer MSUs cannot maximize recall. However, as long as *N* is neither too small nor close to the number of all MSUs, we should see similar results. We examine this by calculating classification performance for various *N*.

#### 4.1.3. Statistical Analysis

We calculate 95% percent confidence intervals for precision, recall, and F-score via statistical bootstrapping (Davison and Hinkley, 1997), with each bootstrap based on 100 random samples with replacement, and a sample size equal to the number of data points.

For example, consider a chunk set of size *N* = 10, 000, selected from the 50,199 MSUs in the BE corpus. In this case, each of the 10,000 MSUs included in the chunk set is assigned a *word* label, and the remaining 40,199 MSUs are labeled as *non-words*. To bootstrap confidence intervals for the three classification metrics, we first take a random sample (with replacement) of 50,199 MSUs (all available data points). Next, we calculate precision and recall for this sample, based on the labels assigned during the classification step. By repeating this procedure 100 times, we obtain a normal distribution of classification metrics—and their 95% confidence intervals correspond to the range between the 2.5th and the 97.5th percentiles. When comparing metrics derived from two different chunk sets, we bootstrap 95% confidence intervals for the difference between them. If zero is not contained within this interval, we can claim with 95% certainty that the difference is not due to chance.

### 4.2. Results and Discussion

**Figure 1:**
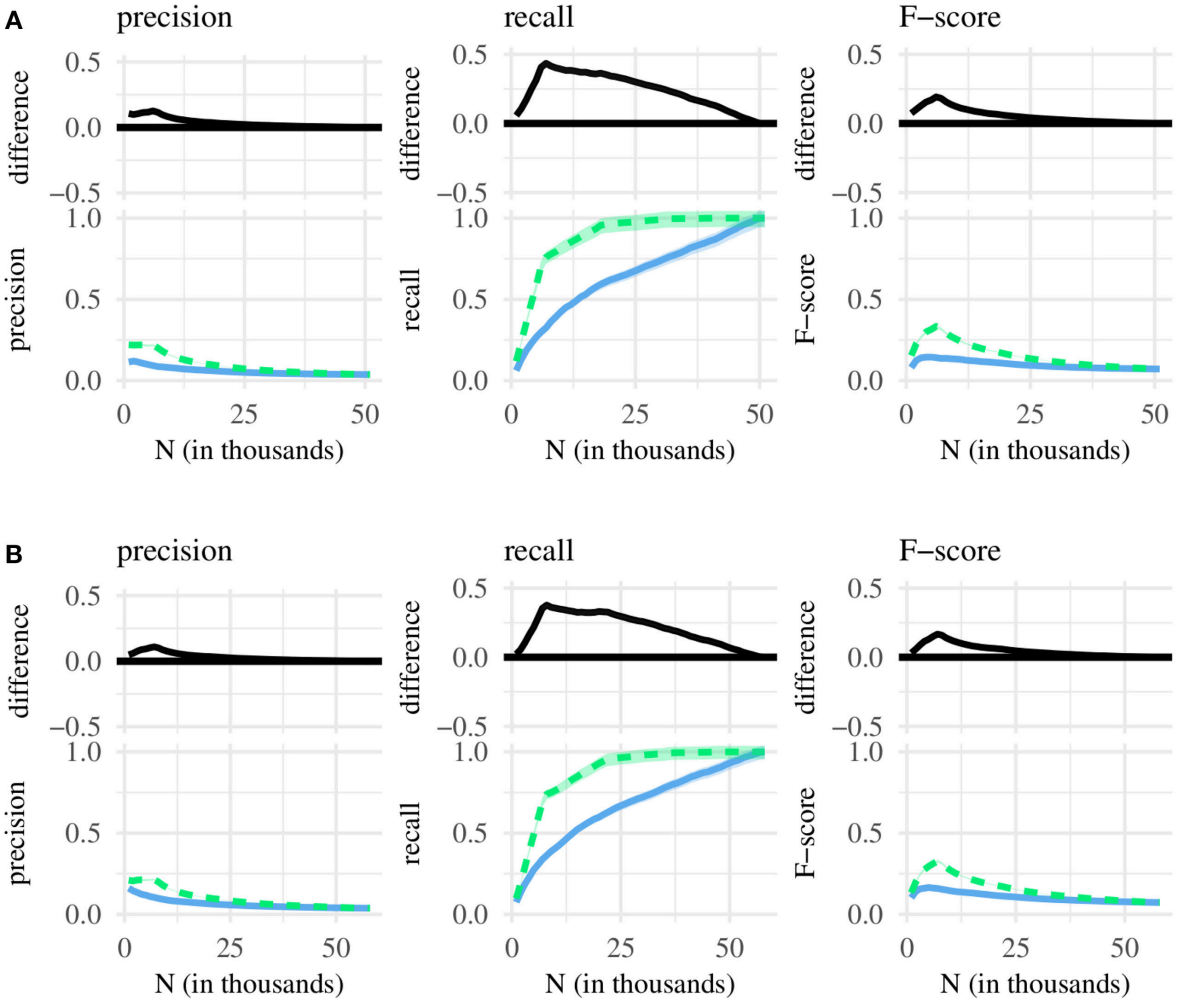
Bottom of each subplot: classification performance for the *N* shortest (green line) and *N* most frequent MSUs (blue line), with 95% confidence intervals. Top: difference between green and blue line, with 95% confidence intervals. **(A)** Chunk sets taken from the BE corpus. **(B)** Chunk sets taken from the NA corpus.

**Figure 2:**
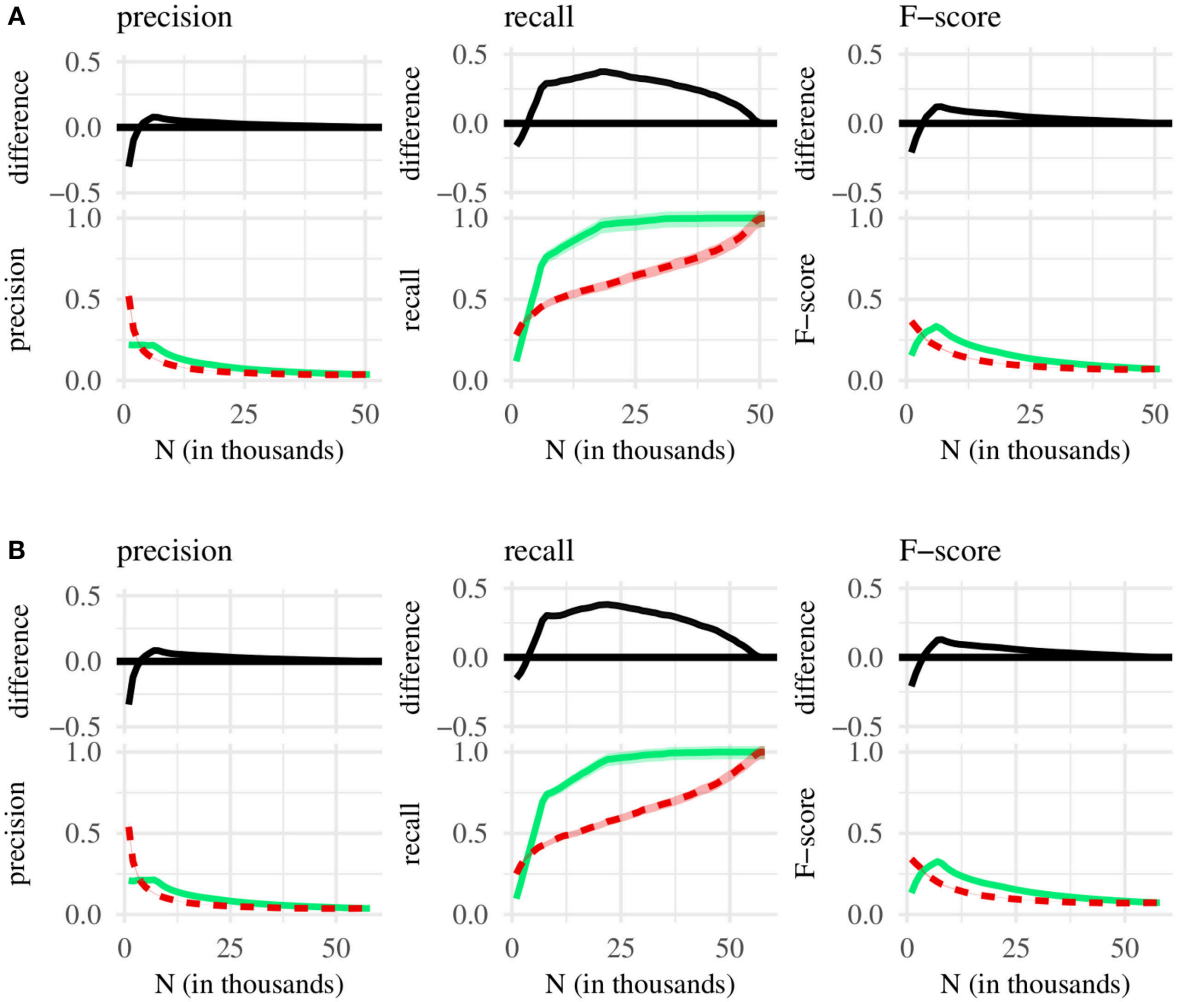
Bottom of each subplot: classification performance for the *N* shortest (green line) and *N* most internally predictable MSUs (red line), with 95% confidence intervals. Top: difference between green and red line, with 95% confidence intervals. **(A)** Chunk sets taken from the BE corpus. **(B)** Chunk sets taken from the NA corpus.

**Figure 3:**
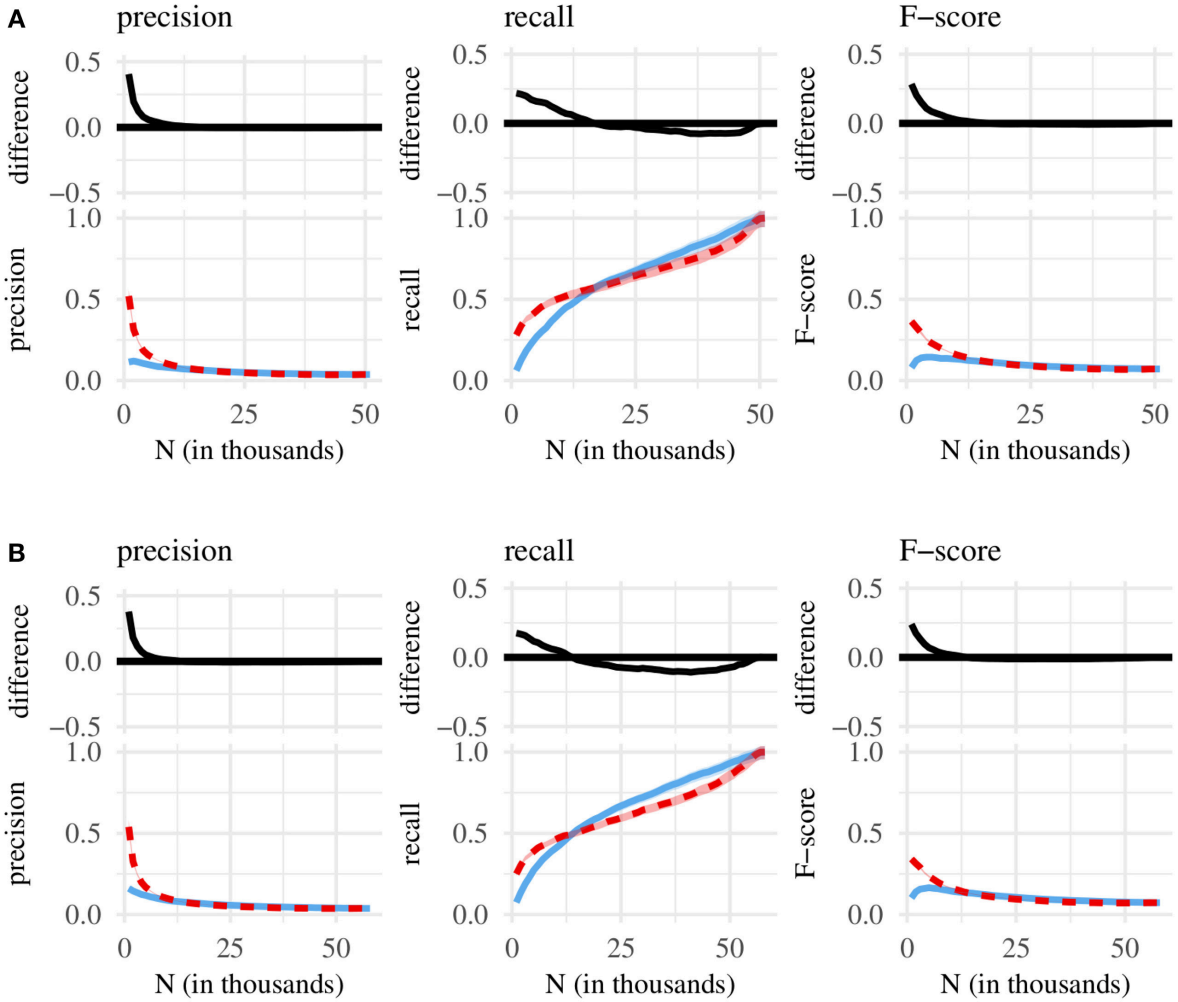
Bottom of each subplot: classification performance for the *N* most internally predictable (red line) and *N* most frequent MSUs (blue line), with 95% confidence intervals. Top: difference between red and blue line, with 95% confidence intervals. **(A)** Chunk sets taken from the BE corpus. **(B)** Chunk sets taken from the NA corpus.

We compare classification metrics associated with three different chunk sets—containing either the shortest, the most frequent, or the most internally predictable MSUs. This design yields three pairwise comparisons of chunk sets: (1) shortest vs. most frequent, (2) shortest vs. most predictable, and (3) most predictable vs. most frequent—each conducted for three metrics of classification performance (precision, recall, F-score), using chunk sets taken from two corpora of English CDS (the BE and the NA corpus). The comparisons are summarized, in turn, by Figures 1–**3** below. Each figure plots, as a function of *N*, classification performance for two different chunk sets, as well as the difference between performance scores. On the x-axis, we increment *N* in steps of 1,000—beginning at *N* = 1, 000 and ending at the maximum possible chunk set size.

Figure 1 shows classification performance for chunk sets containing the *N* shortest and the *N* most frequent MSUs. Across both corpora, precision is highest at *N* = 1, 000, where it is just above 0.2 for the shortest and between 0.1 and 0.15 for the most frequent MSUs. That is, ca. 20% of the shortest 1,000 MSUs correspond to single words, while the same is true for only 10–15% of the most frequent MSUs. Precision then decreases with an increasing chunk set size—to about 15% and 8% at *N* = 10, 000, and to ca. 7% and 4% at *N* = 25, 000. At *N* = 50, 000, the two chunk sets each contain almost all available MSUs, so precision scores derived from either set are very close to one another. However, until the chunk sets contain approximately half of the available MSUs, precision is clearly higher for the *N* shortest MSUs, with the scores approaching each other as *N* is further increased.

Recall increases rather than decreases over successive chunk set sizes. This is because recall can be maximized, at the cost of low precision, by assigning the *word* label to every MSU. Thus, at *N* = 50, 000, recall is close to 1.0 for both chunk sets (i.e., they each contain close to 100% of single-word MSUs) simply because they contain almost all available MSUs, while precision is close to zero (i.e., the proportion of selected single-word MSUs is very low). Conversely, at *N* = 1, 000, recall is minimized, while precision is maximized. Thus, smaller chunk sets contain a large proportion of words, but the majority of single-word MSUs remains undetected. Crucially, with the exception of chunk sets close to the maximum possible size, recall is generally higher for short rather than frequent MSUs.

The harmonic mean of precision and recall (F-score) is maximized at *N*≈10, 000 (short MSUs ≈ 0.25; frequent MSUs ≈ 0.15). Generally, chunk sets containing short rather than frequent MSUs translate into significantly higher F-scores. The difference begins to disappear at around 25, 000, reflecting the fact that as we increase the size of chunk sets, the MSUs contained within them tend to overlap more. As long as we focus on small *N*, however, chunk sets containing short MSUs are clearly better-suited for selecting words.

When comparing short to internally predictable MSUs (Figure 2), we find that very small chunk sets with predictable MSUs (*N* = 1, 000 and *N* = 2, 000) contain a noticeably larger amount of single-word MSUs than equally sized chunk sets with frequent MSUs (50% vs. 10–15% at *N* = 1, 000; 20% vs. 12–15% at *N* = 2, 000). At *N* = 1, 000, predictable MSUs also yield slightly better recall; but almost all subsequent chunk sets capture a much larger proportion of the available single-word MSUs if they are selected according to syllable length rather than predictability. This is reflected in the F-score, which is significantly higher for short MSUs, as long as *N* is not too small or too large. Generally, then, chunk sets containing short rather than predictable MSUs tend to be better-suited for selecting utterances corresponding to individual words.

The only exception to this comes in the form of the 1,000–2,000 most predictable MSUs, which tend to be words more often than their counterparts in equally sized chunk sets with short MSUs. One possible explanation for this pattern is that our predictability metric picks up on low-frequency words, with syllables that occur in only a handful of syllabic contexts, leading to relatively high conditional probabilities for MSUs containing such syllables. But the most predictable MSUs include both low-frequency words (*e.g., vampire, husband, costume*), as well as more common words such as *hello* or *brilliant* (cf. Table 3). Moreover, the average frequency of the 1,000 most predictable MSUs (62 in the BE corpus, 67 in the NA corpus) is actually *higher* than the average frequency of the 1,000 shortest MSUs (26 in the BE corpus, 15 in the NA corpus)—demonstrating that highly predictable MSUs are not all low-frequency items.

In the last remaining comparison (predictability vs. frequency, Figure 3), the high classification performance of small sets containing predictable MSUs exceeds the performance associated with (small) sets of frequent MSUs. Unlike MSU length, whole-sequence frequency is not associated with particularly high recall scores—and larger chunk sets containing frequent MSUs perform, at best, only slightly better than (larger) chunks sets of predictable MSUs. On the whole, predictability thus wins out over frequency.

Syllable length—chiefly due to high recall—in turn won out over predictability (Figure 2) and clearly lead to better performance than frequency (Figure 1). Ordered from worst to best classification performance, that is, we obtain the following ranking: (1) frequency, (2) syllabic predictability, (3) length in syllables. Of course, a small number of constituent syllables does not guarantee that a given MSU will in fact correspond to a single word. But by and large, selections of short MSUs are better-suited for picking out single-word utterances than either frequent or internally predictable MSUs.

This verifies our initial assumption that whole-sequence frequency is a poorer indicator of wordhood than either sequence length or syllabic predictability. In the following analysis, we investigate which of the three MSU types are most likely to be stored, during early speech segmentation, as undersegmented chunks.

## 5. Analysis III: Which Multi-Syllable Utterances Best Predict the Age of First Production of Words?

In the previous analysis, we examined whether short, frequent, or predictable MSUs are more likely to correspond to single words. Now, we evaluate how well the three different types of MSUs predict the age at which their component words are acquired, arguing that MSUs which are well-suited for predicting word learning are also more likely to be stored as undersegmented chunks. Since frequency of occurrence seems to confer a general learning advantage (Ambridge et al., 2015), children might preferentially store frequent MSUs as chunks. It is also possible, however, that children are biased to extract and store more discrete, word-like MSUs. If true, we should expect children to store short and possibly internally predictable MSUs, to the exclusion of more frequent items.

### 5.1. Method

Following Grimm et al. (2017), we use the MSUs in a particular chunk set to predict the age at which children first produce the words contained within the MSUs. Grimm et al. (2017) found that words which are contained in a large number of multi-word phrases are produced at earlier stages than words contained in fewer phrases. As a possible explanation, they argued that children commit phrases to long-term memory as holistic chunks—i.e., before they have discovered that the phrases are composed of smaller linguistic units. As a result, the more chunks containing a particular word *X* are stored in long-term memory, the higher the likelihood that children discover *X* as a separate linguistic unit—and the earlier they subsequently produce *X*. We thus evaluate how well the MSUs from different chunk sets perform at predicting the age of first production (henceforth *AoFP*) of their component words. If children store frequent MSUs as chunks—prior to having detected the words contained within those chunks—, then frequent MSUs should perform best. Conversely, if they store short or internally predictable MSUs, short or predictable MSUs should perform best.

We implement this idea by using AoFP as a dependent variable in multiple linear regressions. Given a chunk set and a set of words with associated AoFP values (henceforth *target words*), we count—for each target word—how many MSUs within the chunk set contain it. The resulting value, the number of MSUs per target word (henceforth *#MSU*), is then used as an independent variable. We denote this measure *#MSU*-*F* when calculated based on the *N* most frequent MSUs, *#MSU*-*S* when calculated based on the *N* shortest MSUs, and *#MSU*-*P* when calculated based on the *N* most predictable MSUs. Thus, by using *#MSU*-*F*, *#MSU*-*S*, and *#MSU*-*P* to predict AoFP, we evaluate how well the shortest, most frequent, and most predictable MSUs perform at predicting the time course of word learning. If children store short MSUs as chunks, then *#MSU*-*S* should perform best at predicting AoFP; if they store frequent MSUs, *#MSU*-*F* should perform best; and if they store predictable MSUs, *#MSU*-*P* should emerge as the best-performing predictor.

To evaluate performance, we track two statistics: (1) the regression coefficient (β), measuring how strongly the AoFP of targets decreases as we increase *#MSU*; and (2) the amount of variance within AoFP that can be accounted for by including *#MSU* in the regression models (*R*^2^). We expect that a robust result should lead to comparable effects across the two statistics. For example, if words contained within predictable MSUs are learned earlier than words contained within frequent or short MSUs, words with high *#MSU*-*P* counts should be learned earlier than words with high *#MSU*-*F* or *#MSU*-*S* counts—and this should be reflected in stronger effects, across the two statistics, for *#MSU*-*P*.

#### 5.1.1. Age of First Production

Selecting suitable AoFP data is critical, as the procedure used to obtain AoFP estimates could confound the results. Specifically, children might produce chunks without having learned about the words within them. We should make sure, in other words, that AoFP estimates are based on word productions which are not performed in the context of the MSUs used to predict AoFP. We control for this in the first of two AoFP data sets, which we estimate from the children addressed in the two CDS corpora. And to ensure the robustness of these corpus-derived AoFP estimates, we replicate our results on an existing data set derived from parent-report questionnaires.

##### 5.1.1.1. Corpus-derived AoFP

The first AoFP data set is estimated from the transcribed speech of the children addressed by the caregivers in the two aggregated CHILDES corpora^10^. Here, we treat a word as having been acquired at the earliest developmental stage at which any child within a corpus produces it. In doing so, we only consider word productions from outside of (any and all) adult-produced MSUs. For example, if a child produces the word *day* as part of the adult-produced MSU *what a great day*,we do not consider the child production. Further, we do not consider word productions if the words are produced within sub-sequences of adult-produced MSUs (with the exception of single-word sub-sequences, i.e., target words produced in isolation). We would not, that is, consider child productions like *it's a great day*, since *a great day* is a sub-sequence of *what a great day*. Corpus-derived AoFP thus is a conservative estimate, where a given word is considered as learned at the earliest developmental stage at which any child first produces it—in a context without overlap with the adult-produced MSUs^11^.

*Developmental stage* is defined in terms of mean length of utterance (MLU)—the average child utterance length, in tokens, within a transcript (CHILDES corpora consist of transcripts, recorded at different points during the target child's development). We induce MLU rather than AoFP estimates because children who are close in age may nevertheless be far apart in language development. Being a more robust estimator, MLU controls for developmental differences (Parker and Brorson, 2005). Since transcripts contain varying numbers of utterances, the average utterance length per transcript is biased with respect to transcript length. We rectify this issue by estimating MLU for each transcript via statistical bootstrapping (Davison and Hinkley, 1997). Each bootstrap is based on 10,000 random samples with replacement, with the sample size equal to the number of child utterances per transcript. We thus induce MLU rather than AoFP estimates but will, for simplicity, refer to a word's MLU as its AoFP. To calculate an estimate for a given word, we bootstrap the set of MLUs γ for all transcripts within which a child uses the word outside of an adult-produced MSU, and we choose the smallest value in γ as the word's AoFP. Performing this procedure for all words produced by children in at least two of the considered CHILDES corpora, we obtain AoFP estimates for the aggregated BE and NA corpus—covering 7,565 and 9,482 different child-produced words.

##### 5.1.1.2. CDI-derived AoFP

The corpus-derived AoFP estimates are sensitive to high-frequency words, making it desirable to replicate results on data that do not rely on language sampling. We obtain such AoFP estimates from the wordbank database (Frank et al., 2017)^12^, a repository with results from parent-report questionnaires (MacArthur–Bates Communicative Development Inventories / CDI). Wordbank archives data from various administrations of the CDI. The largest English data set pools responses from parents of 6,945 (American) English-speaking children between the ages of 16 and 30 months and covers 680 words and phrases.^13^ At each of the 15 months covered by the questionnaires, parents had to indicate whether their child produces a list of words. Word-level data are then represented as the percentage of parents who reported, for a given month, that their child can successfully produce the word in question. Excluding compounds, phrases, and words that are specific to particular children (baby sitter's name, child's own name, pet's name), we derive AoFP estimates for 647 words by counting words as having been learned if at least 50% of the children were reported to produce it. Due to the design of the CDI, we cannot rule out that parents reported on child productions of chunks instead of individual word productions. Corpus-derived AoFP, which controls for chunk productions, is thus of primary importance. And to increase confidence in the robustness of results, CDI-derived AoFP is used to replicate results achieved with the former.

Since the children whose parents filled in the CDI forms were no older than 30 months, we restrict the MSUs included in chunk sets for the analyses with CDI-derived AoFP—considering only MSUs which were produced in the presence of children aged 30 months or less.

#### 5.1.2. Validity of AoFP Estimates

It would raise methodological concerns if we simply assumed the validity of corpus-derived AoFP. The CDI-derived estimates, on other hand, have been validated on different measures of children's expressive vocabularies (Dale, 1991; Fenson et al., 2007). This is why we include CDI-derived estimates, and why it is important that similar results are obtained with both data sets. To further increase our confidence in both types of estimates, we compare them to the only publicly available English age of acquisition estimates that come directly from children: Morrison et al. (1997) had children of varying ages perform a picture naming task; and if a child was able to produce the correct picture name, he or she was considered to have acquired the word.

Presumably because of time constraints, Morrison et al. (1997) provide age of acquisition for a restricted set of 297 picturable nouns. While insufficient for our analyses, we can still use their data to verify our estimates: The correlation between their estimates and corpus-derived AoFP is strongly positive (Spearman's *rho* = 0.65 for the BE children and *rho* = 0.59 for the NA children, based on 274 and 272 shared words, respectively; *p* < 10^−8^). The correlation with CDI-derived AoFP is also fairly strong (*rho* = 0.50, based on 117 shared words, *p* < 10^−8^). This pattern strengthens our confidence in the validity of (corpus- and CDI-derived) AoFP estimates.

#### 5.1.3. Co-variates

The independent variable is *#MSU*, which we use to predict AoFP. Grimm et al. (2017) found that a similar predictor is negatively correlated with AoFP, leading us to also expect a negative correlation between *#MSU* and AoFP (meaning that words contained in many MSUs would be learned earlier than words contained in fewer MSUs). But such a correlation could be due to collinearity with several co-variates, the most obvious of which is word frequency. Frequency of exposure is associated with a general learning advantage (Ambridge et al., 2015), and words with a high *#MSU* count tend to be frequent. Grimm et al. (2017) controlled for frequency, but there are other possible confounds.

We attempt to remedy this by including the following co-variates: (1) the corpus frequency, in CDS, of each target word (*Freq*), (2) concreteness ratings (*Con*), (3) length in syllables (*Nsyl*), and (4) phonological neighborhood density (*PhonN*)^14^. *Freq* must be included to control for frequency of exposure, and *Con* is included to control for semantic properties of target words. *Nsyl* and *PhonN*, meanwhile, are meant to control for confounds having to do with the phonological properties of target words. Concreteness ratings for 40,000 lemmas are taken from Brysbaert et al. (2014)^15^, who collected them from over 4,000 participants via Mechanical Turk. Since ratings were collected for lemmas, we assigned the lemma rating to all its word forms. Given a target word, *PhonN* is defined as the number of homophones, plus the number of words that can be derived from the target by either adding, deleting, or substituting a single phoneme. *PhonN*, together with *Nsyl*, is derived from the syllabified CMU pronouncing dictionary that was also used to convert our corpora to syllable representations. Braginsky et al. (2016) have recently shown that variables similar to *Freq, Con*, and *Nsyl* predict age of acquisition: Early-acquired words tend to be frequent, concrete, and (at least in English) short. We additionally include *PhonN*, as words in dense neighborhoods tend to be early-learned, possibly due to a memory advantage of highly connected lexical representations (Storkel, 2004, 2009). Below, we report analyses for regression models that include the co-variates. Results without covariates are reported in the Supplementary Material (Appendix H). Appendix G additionally controls for the age at which children are first exposed to MSUs.

#### 5.1.4. Statistical Analyses

When working with the corpus-induced AoFP data, we use AoFP estimates from children who were not addressed in the corpus used to calculate *#MSU*. In other words, we use AoFP from the children addressed in the NA corpus for regression models which include *#MSU* and frequency counts from the BE corpus; and we use AoFP from the children addressed in the BE corpus for regression models which include independent variables from the NA corpus. This design de-couples the independent variable from corpus-induced AoFP and is meant to increase the generality of our study's implications. Since the CDI-derived AoFP estimates come from an external source, we use MSUs from both the BE and NA corpus to predict the CDI data—although restricted, as mentioned, to MSUs produced in interactions with children aged 30 months or less.

This leaves us with three different corpus-AoFP pairings: (1) BE corpus with AoFP from NA children, (2) NA corpus with AoFP from BE children, and (3) age-restricted BE and NA corpus with CDI-derived AoFP. The corpus material used in each analysis contains around 50,000 MSUs. Regression analyses are based on all words for which *PhonN, Nsyl, Con*, and AoFP estimates are available: 6,208 and 5,577 words for analyses (1) and (2), and 615 words for analysis (3). Each data set contains AoFP values for content as well as function words. Additional information on the target words is presented in the Supplementary Material (Appendix D).

In order to avoid problems from zero counts, *#MSU* was increased by 1. All variables were log-transformed and then standardized (via transformation to Z-scores). We compute 95% percent confidence intervals for regression coefficients and *R*^2^ values via statistical bootstrapping (Davison and Hinkley, 1997), with each bootstrap based on 100 random samples with replacement, and a sample size equal to the number of data points. When comparing two effects, we bootstrap 95% confidence intervals for the difference between them. If zero is not contained within this interval, we can claim with 95% certainty that the difference is not due to chance.

### 5.2. Results and Discussion

**Figure 4:**
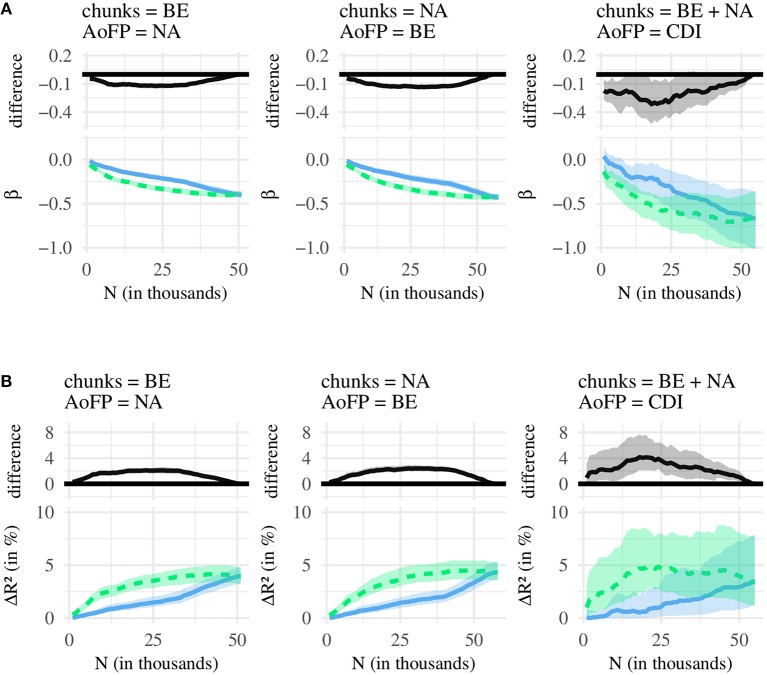
Comparison of *#MSU*-*S* (green line) and *#MSU*-*F* (blue line). **(A)** Bottom: regression coefficients (β), with 95% confidence intervals. Top: difference between green (*#MSU*-*S*) and blue (*#MSU*-*F*) line, with 95% confidence intervals. **(B)** Bottom: amount of variance in AoFP (Δ*R*^2^ in %), with 95% confidence intervals, that can be explained by *#MSU*. Top: difference between green (*#MSU*-*S*) and blue (*#MSU*-*F*) line, with 95% confidence intervals.

**Figure 5:**
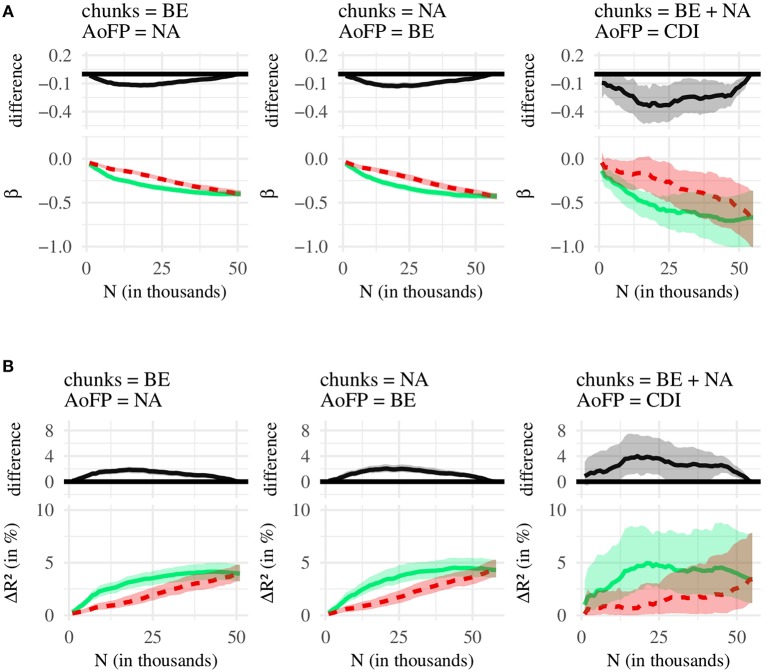
Comparison of *#MSU*-*S* (green line) and *#MSU*-*P* (red line). **(A)** Bottom: regression coefficients (β), with 95% confidence intervals. Top: difference between green (*#MSU*-*S*) and red (*#MSU*-*P*) line, with 95% confidence intervals. **(B)** Bottom: amount of variance in AoFP (Δ*R*^2^ in %) that can be explained by *#MSU*, with 95% confidence intervals. Top: difference between green (*#MSU*-*S*) and red (*#MSU*-*P*) line, with 95% confidence intervals.

**Figure 6:**
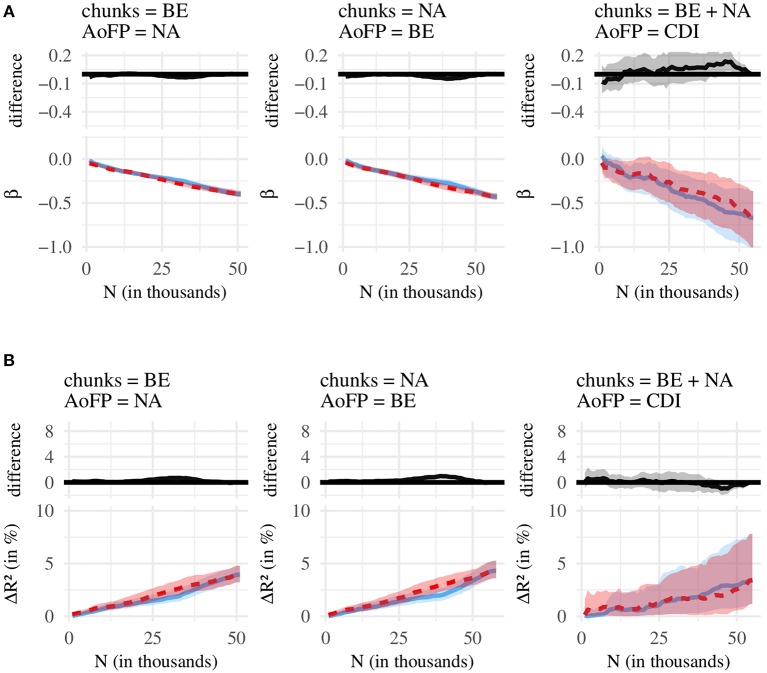
Comparison of *#MSU*-*P* (red line) and *#MSU*-*F* (blue line). **(A)** Bottom: regression coefficients (β), with 95% confidence intervals. Top: difference between red (*#MSU*-*P*) and blue (*#MSU*-*F*) line, with 95% confidence intervals. **(B)** Bottom: amount of variance in AoFP (Δ*R*^2^ in %) that can be explained by *#MSU*, with 95% confidence intervals. Top: difference between red (*#MSU*-*P*) and blue (*#MSU*-*F*) line, with 95% confidence intervals.

We compare the effects associated with three independent variables (*#MSU*-*S*, *#MSU*-*F*, *#MSU*-*P*), resulting in three pairwise comparisons: (1) *#MSU*-*S* vs. *#MSU*-*F*, (2) *#MSU*-*S* vs. *#MSU*-*P*, and (3) *#MSU*-*P* vs. *#MSU*-*F*. Each of these comparisons is conducted for two statistics (β, *R*^2^) and three corpus-AoFP pairings (calculate *#MSU* from BE corpus and AoFP from NA corpus; calculate *#MSU* from NA corpus and AoFP from BE corpus; calculate *#MSU* from age-restricted NA plus BE corpus and use CDI-derived AoFP). Figure 4 summarizes the first set of comparisons, for (1) *#MSU*-*S* vs. *#MSU*-*F*. Figure 5 then summarizes (2) *#MSU*-*S* vs. *#MSU*-*P*, and Figure 6 summarizes (3) *#MSU*-*P* vs. *#MSU*-*F*. We discuss each comparison in turn.

Figure 4A shows, as a function of *N*, the regression coefficients for *#MSU*-*S* and *#MSU*-*F*, as well as the difference between both; and Figure 4B does the same for *R*^2^. Similar to the plots presented in the previous analysis, each figure begins with *N* = 1, 000, which is then incremented in steps of 1,000 until *N* is equal to the number of all available MSUs. For most *N*, the coefficient for *#MSU*-*S* is more strongly negative than the coefficient for *#MSU*-*F*. Thus, the more MSUs contain a given word, the earlier that word is first produced, and this predictive relationship is stronger for *#MSU*-*S* than for *#MSU*-*F*. We find a similar pattern for *R*^2^: Across most *N*, *#MSU*-*S* can explain a larger amount of variance in AoFP than *#MSU*-*F*. We can state, then, that *#MSU*-*S* performs better at predicting AoFP.

This pattern is similar across all three pairings of corpus and AoFP data, although the confidence intervals are much larger when using CDI-derived AoFP. This is probably due to the smaller number of data points: The regression models with CDI estimates are based on 615 words, while the regressions with corpus-derived estimates include approximately ten times the number of words. As a result, we operate with less statistical power when conducting analyses with the CDI-derived estimates, and the differences between β / *R*^2^ do not always reach statistical significance. The overall pattern, however, is similar across the different AoFP estimates—indicating that *#MSU*-*S* is indeed better-suited for predicting AoFP.

The only choices of *N* for which this is not true are (a) very small values and (b) values close to the largest possible value. Generally, β and *R*^2^ take near-zero values at *N* = 1, 000. This is because at 1,000 MSUs, we can only derive *#MSU* counts for a relatively restricted number of target words. But as we increase *N*, *#MSU*-*S* and *#MSU*-*F* begin to perform better. *R*^2^ increases, and the coefficients associated with the two predictors now take negative values. Crucially, regression models with *#MSU*-*S* outperform their counterparts with *#MSU*-*F*.

At some point, the difference starts to decrease, until it disappears once *N* is equal to the number of all MSUs. This makes sense: If the two chunk sets contain all MSUs, *#MSU*-*S* and *#MSU*-*F* are calculated from the same selection of MSUs, and the two estimates will take the same value. A larger *N* means that the two chunk sets from which we calculate *#MSU*-*S* and *#MSU*-*F* overlap more and more, and the two estimates begin to converge. Thus, past a certain point, the differences in *R*^2^ and β decrease.

We thus have good reason to claim that *#MSU*-*S* is better suited for predicting the time course of word learning than *#MSU*-*F*. Figure 6 shows, moreover, that *#MSU*-*S* also outperforms *#MSU*-*P*, with a pattern that is very similar to the one obtained in the previous comparison. At the same time, almost no significant difference emerges when comparing *#MSU*-*P* and *#MSU*-*F* (Figure 6). Together, the three comparisons suggest that there is no (strong) difference in the effects obtained with *#MSU*-*P* and *#MSU*-*F*, while *#MSU*-*S* performs consistently better at predicting AoFP than the other two *#MSU* counts.

The effect size, however, is rather small: Baseline models that include the covariates explain approximately 25–45% of the variance in AoFP (see the Supplementary Material, Appendix E), while the addition of *#MSU*-*S* only increases this by 4–5% (given a sufficiently large chunk set). In addition, a *post-hoc* analysis revealed that chunk sets containing the 10,000 shortest MSUs also cover a larger proportion of target words (by about 10 percentage points) than corresponding chunk sets with particularly frequent or internally predictable items (see the Supplementary Material, Appendix F). Thus, *#MSU*-*S* might explain more variance in AoFP than the other two predictors simply because there is a larger proportion of target words with non-zero *#MSU*-*S* counts, relative to *#MSU*-*F* and *#MSU*-*P* counts.

The larger coverage of target words may thus bias results with respect to the amount of variance that can be explained in AoFP. Note, however, that *#MSU*-*S* is *also* associated with a more strongly negative regression coefficient, which is unrelated to the number of data points (provided that there is a bare minimum of data points). We can thus still claim that particularly short chunks are better-suited for predicting word learning than either especially frequent or internally predictable chunks—albeit by a small margin. With these caveats, the results suggest that children may not store many complete utterances as undersegmented chunks; but that when they do store chunks, these are more likely to correspond to short rather than frequent or internally predictable MSUs.

## 6. General Discussion

In this paper, we compared (1) frequent, (2) short, and (3) internally predictable chunks. In one of two analyses, we found that selections of short MSUs tend to contain more single-word utterances than selections of frequent or predictable MSUs, suggesting that sequence length is a more useful cue to *wordhood* than the other two predictors. In a second analysis, we also found that short MSUs perform better at predicting the time course of word learning. Together, the two analyses suggest that undersegmented chunks, to the extent that they are stored by children, tend to be short and word-like sequences—rather than frequent or internally predictable multi-word chunks.

We hypothesize that the results are partly due to children's memory constraints, an argument that forms part of item-based learning (MacWhinney, 1978, 2014). Within this theoretical framework, memory constraints are assumed to prevent children from storing longer speech sequences. Instead, children are thought to extract relatively short sequences (e.g., short phrases or multi-morpheme words) as unsegmented units. These sequences are then further segmented via comparison to known items. Some of the units discovered in this manner will correspond to predicates, which children relate to particular arguments in the context of item-based patterns (e.g., *his + object*). Thus, the claim is that part of children's early lexical and syntactic development can be traced back to *short* input sequences. From this perspective, it is not surprising that short MSUs outperform frequent and internally predictable MSUs at predicting the time course of word learning.

Given that the effect size is relatively small (short chunks only explain 4–5% of the variance in AoFP), children may not store many complete utterances as undersegmented chunks. Since we also found, in analysis II, that short MSUs are the most word-like, our findings instead support a scenario wherein most of the units which children extract as hypothesized words correspond to *actual* words. Given MacWhinney (2014)'s finding that approx. 25% of (English) parental utterances are single-word utterances, child-directed speech would appear to be well-suited for supporting such a segmentation strategy.

Children's memory constraints, then, might result in a segmentation bias toward discrete or indivisible linguistic units—i.e., word-like sequences that cannot themselves be segmented into smaller units. In addition, children might occasionally extract and store short multi-morpheme chunks, driving lexical and syntactic learning (MacWhinney, 1978, 2014). Such an account can explain our results, and it also lines up with the nature of children's early productions—which consist mostly of single-word utterances,^16^ despite occasional productions of apparently undersegmented material, as reported by e.g., Peters (1983).

This perspective has implications for research concerned with frequent multi-word sequences, which are sometimes referred to as *formulaic sequences*. Various studies have demonstrated that both adults (Arnon and Snider, 2010; Arnon and Priva, 2014) and children who have completed the segmentation process (Bannard and Matthews, 2008; Arnon and Clark, 2011) are faster to process formulaic multi-word phrases, and that this processing advantage cannot be reduced to the frequency of individual words. Such results suggest that language users represent some aspect(s) of frequent word sequences—above and beyond information about constituent words.

Since the subjects in these studies had completed the segmentation process, it is unlikely that they process multi-word phrases in a holistic fashion, without accessing component words. Indeed, other studies have collected evidence that access (in adult processing) to frequent trigrams (Arnon and Priva, 2014), to idioms (Sprenger et al., 2006), and to frequent adjective-noun and noun-noun phrases (Jacobs et al., 2016) involves access to individual words. Post-segmentation, that is, language users appear to possess analyzed representations of multi-word phrases. This naturally leads to the question whether holistically stored chunks are retained past the segmentation stage as fully analyzed representations, or whether chunks are discarded once the segmentation process is completed. In the latter case, chunks and representations of frequent phrases would result from two different processes. One would be related to segmentation and involve the storage of larger units that are gradually analyzed, and the other would discover phrases through usage patterns within fully segmented input (Arnon and Christiansen, 2017).

The results presented in this study imply that children preferentially store word-like sequences as undersegmented chunks—which tend to be short, not frequent. This, in turn, supports accounts wherein representations for frequent multi-word sequences tend to emerge *after* the segmentation process has run its course. Arguing from the current results, in other words, we suggest that most cognitive representations for formulaic multi-word sequences cannot be traced back to undersegmented chunks in children.

## 7. Limitations and Open Questions

We have presented results, from an exploratory study, intended to inform research on undersegmented chunks in child language acquisition. These results were obtained after imposing two filters on the chunks used to predict child word learning: (1) Chunks were required to be at least two syllables long, and (2) each chunk had to occur in at least two of the considered CHILDES corpora. In addition, our results are correlational in nature, and our interpretation may could be confounded by one or more unknown variables. We address both concerns below.

The two filtering steps, while undesirable, were necessary given the design of our study. We excluded single-syllable sequences from our analyses since these are already fully segmented (given our assumption about the primacy of proto-syllables during language development)—whereas we are interested in unsegmented chunks. Future work should explore possible sub-syllabic perceptual primitives (e.g., phones), which would allow us to treat monosyllabic utterances as unsegmented units.

We also excluded multi-syllable chunks that occurred in only one out of the 51 considered CHILDES corpora. By collapsing data from many different corpora, we attempted to leverage the large amount of English child-directed speech contained in the entire CHILDES data base. However, if we had considered all chunks from the 51 CHILDES corpora, we would have selected many chunks that are idiosyncratic to particular child-caregiver dyads—i.e., chunks that are not contained in most children's input. In other words: We would have used very rare chunks to predict when children *generally* learn to use words, even though most children will never have an opportunity to acquire words in the context of these exceedingly rare chunks. To alleviate this problem, we imposed a minimum count of two CHILDES corpora per chunk. Future work should implement a longitudinal design, which would remove the need for this filtering step.

Lastly, given that ours is a correlational study, there may be several different causes that could explain the results. We have tried to exclude confounding variables by controlling for a number of covariates, as well as by ensuring that child productions of target words are not due to children simply repeating the chunks we used to predict word learning. Nevertheless, causality can only be established through experiments with human participants. A possible direction for future work is to test the predicted segmentation bias for short sequences in an artificial word segmentation task that contrasts short with frequent and internally predictable speech sequences.

## Author Contributions

RG conceived of the original conceptual framework and design of this study and drafted the manuscript. GC was involved in critically revising and adding to the design, helped with the analysis, and made important additions to the manuscript. SG and WD contributed critically to both the conception and design of this work and crucially revised the manuscript. All authors approved the final version for publication and agree to be accountable for all aspects of the work as well as to ensure that questions related to the accuracy or integrity of any part of the work are appropriately investigated and resolved.

### Conflict of Interest Statement

The authors declare that the research was conducted in the absence of any commercial or financial relationships that could be construed as a potential conflict of interest.

## Footnotes

^1^Both examples were originally reported by Clark (1974).

^2^This idea re-surfaces in accounts of adult linguistic competence which include constructions as constituents (Goldberg, 2006; O'Donnell, 2015).

^3^In psycholinguistics, the term *transitional probability* has come to denote conditional probabilities between units. Conditional probability is a measure of association strength between two elements that is normalized by the frequency of the non-conditional element.

^4^Various studies have since shown that the underlying mechanism can operate on non-linguistic auditory as well as visual stimuli, and that it is not restricted to humans. See Aslin (2017) for a review.

^5^See Phillips and Pearl (2015) for similar models which operate on syllables rather than on phonemes.

^6^Cf. Phillips and Pearl (2015), who compared several state-of-the-art Bayseian segmenters to Lignos (2012)'s model. As long as the input is represented in terms of syllables, and not in phonemes, the chunk-based segmenter performs similarly to Bayseian approaches.

^7^See the Supplementary Material, Appendix A, for a list of included corpora.

^8^http://webdocs.cs.ualberta.ca/~kondrak/cmudict.html

^9^For readability, each MSU is presented in its orthographic transcription. But note that in our experiments, MSUs are represented as unsegmented syllable sequences. For example, the orthographic transcription *that's wonderful* is underlyingly represented as *thats1-won1-der0-ful0* (1 = primary stress, 2 = secondary stress, 0 = no stress).

^10^See the Supplementary Material, Appendix B, for additional information on the corpus material used to estimate AoFP.

^11^This way of measuring first productions is extremely conservative and could potentially confound the results. For this reason, we made sure that results of the regression analyses, reported below, are similar if AoFP is simply defined as first use. We also made sure that the different AoFP data sets are strongly correlated with one another (see correlation coefficients reported below).

^12^Available online: http://wordbank.stanford.edu/

^13^Data were downloaded on 01/08/2018.

^14^See the Supplementary Material, Appendix C, for a collinearity analysis.

^15^http://crr.ugent.be/archives/1330

^16^Cf. standard text books on first language acquisition, e.g., Clark (2009).
