# Supplementary material for: Children Probably Store Short Rather Than Frequent or Predictable Chunks: Quantitative Evidence From a Corpus Study
Source: Front Psychol. 2019 Jan 30;10:80. doi: 10.3389/fpsyg.2019.00080 (PMC6363945; doi:10.3389/fpsyg.2019.00080)
Supplement: Supplementary file 1 [file Data_Sheet_1.PDF]

APPENDIX A: CHILDES CORPORA

The aggregated corpora of transcribed adult- and child-produced speech used in this study contain material from all (British and North American) English corpora on the CHILDES data base (MacWhinney, 2000a) where the target children were normally developing (available online: `https://childes.talkbank.org/`). The BE corpus is based on ten CHILDES corpora, and the NA corpus is based on 41 CHILDES corpora (data were downloaded August 2018).

1. CHILDES corpora used for the BE corpus:

   Belfast (Henry, 1995), Fletcher (Fletcher and Garman, 1988), Forrester (Forrester, 2002), Howe (Howe, 1981), Lara (Rowland and Fletcher, 2006), MPI-EVA-Manchester (Lieven et al., 2009), Manchester (Theakston et al., 2001), Thomas (Lieven et al., 2009), Tommerdahl (Tommerdahl and Kilpatrick, 2013), Wells (Wells, 1981)

2. CHILDES corpora used for the NA corpus:

   Bates (Bates et al., 1991), Bernstein-Ratner (Ratner, 1986), Bliss (Bliss, 1988), Bloom 1970 (Bloom et al., 1974), Bloom 1973 (Bloom, 1976), Bohannon (Bohannon III and Marquis, 1977), Braunwald (Braunwald, 1971), Brent (Brent and Siskind, 2001), Brown (Brown, 1973), Clark (Clark, 1978), Cornell (no reference provided), Demetras-Trevor (Demetras, 1986), Evans (no reference provided), Feldman (Feldman and Menn, 2003), Garvey (Garvey and Hogan, 1973), Gathercole (no reference provided), Gleason (Masur and Gleason, 1980), HSLLD (Beals, 1993), Hall (Hall et al., 1984), Higginson(no reference provided), Kuczaj (Kuczaj, 1977), MacWhinney (MacWhinney, 2000b), McCune (McCune, 1995), McMillan (no reference provided), Morisset (Morisset et al., 1995), New England (Ninio et al., 1994), Post (Demetras et al., 1986), Providence (Song et al., 2013), Rollins (Rollins, 2003), Sachs (Sachs, 1983), Sawyer (Sawyer, 1997), Snow (MacWhinney and Snow, 1990), Soderstrom (Soderstrom et al., 2008), Sprott (no reference provided), Suppes (Suppes, 1974), Tardif (no reference provided), Valian (Valian, 1991), Van Houten (Van Houten, 1986), Van Kleeck (no reference provided), Warren-Leubecker (Warren-Leubecker and Bohannon III, 1984), Weist (Weist and Zevenbergen, 2008)

APPENDIX B: CHILD-PRODUCED SPEECH STATISTICS

measure	BE	NA
# children	247	743
mean child age (months)	32.66 (SD = 9.25)	41.39 (SD = 23.45)
# utterances	873,623	846,894
mean utterance length (words)	3.45 (SD = 2.95)	2.51 (SD = 1.88)
# tokens	3,016,863	2,130,946
# types	43,510	24,322

Table 4. Statistics for child-produced speech used to estimate corpus-derived AoFP.

APPENDIX C: CORRELATIONS BETWEEN PREDICTORS

Table 5 shows pairwise correlations between the predictors used in the linear regression analyses. Correlations between $\#MSU$ counts and the co-variates are mostly weak to moderate, but we also find a few stronger correlations (e.g. word frequency is strongly positively correlated with all $\#MSU$ counts). Including collinear predictors in regression models can lead to unstable results. It is thus important that similar results are obtained when the co-variates are excluded. Results for analyses without co-variates are reported in appendix H.

corpus	1. S	2. F	3. P	4. Freq	5. Con	6. Nsyl	7. PhonN
BE	1. —	0.65 ***	0.62 ***	0.69 ***	0.04 ***	-0.24 ***	0.26 ***
	2. —	—	0.69 ***	0.69 ***	-0.07 ***	-0.08 ***	0.14 ***
	3. —	—	—	0.62 ***	-0.02	-0.03 *	0.02 *
	4. —	—	—	—	-0.04 ***	-0.18 ***	0.22 ***
	5. —	—	—	—	—	-0.03 **	0.01
	6. —	—	—	—	—	—	-0.74 ***
NA	1. —	0.64 ***	0.59 ***	0.70 ***	0.03 **	-0.26 ***	0.27 ***
	2. —	—	0.68 ***	0.71 ***	-0.07 ***	-0.08 ***	0.16 ***
	3. —	—	—	0.62 ***	-0.01	-0.01	0.02 *
	4. —	—	—	—	-0.06 ***	-0.20 ***	0.26 ***
	5. —	—	—	—	—	-0.02	-0.01
	6. —	—	—	—	—	—	-0.74 ***
BE + NA	1. —	0.76 ***	0.61 ***	0.81 ***	-0.38 ***	-0.63 ***	0.59 ***
	2. —	—	0.83 ***	0.92 ***	-0.46 ***	-0.25 ***	0.34 ***
	3. —	—	—	0.81 ***	-0.43 ***	-0.11 ***	0.19 ***
	4. —	—	—	—	-0.52 ***	-0.33 ***	0.40 ***
	5. —	—	—	—	—	0.20 ***	-0.18 ***
	6. —	—	—	—	—	—	-0.72 ***

Table 5. Pairwise correlations (Spearman's ρ) for predictors used in regression analyses. S = $\#MSU$-S, F = $\#MSU$-F, P = $\#MSU$-P. ***: $p \leq 0.001$. **: $p \leq 0.01$. *: $p \leq 0.05$.

APPENDIX D: AGE OF FIRST PRODUCTION STATISTICS

part-of-speech	BE	NA	CDI
Nouns	55.0 %	57.0 %	54.0 %
Verbs	24.0 %	23.0 %	19.0 %
Adjectives	12.0 %	12.0 %	0.9 %
Adverbs	0.4 %	0.3 %	0.5 %
Function words	0.5 %	0.4 %	13.0 %
Other	1.0 %	1.0 %	0.0 %

Table 6. Part-of-speech proportions for words in the three different AoFP data sets (AoFP from BE children, AoFP from NA children, CDI-derived AoFP.) Part-of-speech tags correspond to the most frequent part-of-speech for each given word, according to Brysbaert et al. (2014).

AoFP data set	min	max	mean (SD)
BE	1.0	5.54	2.08 (0.73)
NA	1.0	8.31	2.52 (1.05)
CDI	16.0	31.0	24.97 (3.76)

Table 7. Minimum, maximum, and mean AoFP values for the three different AoFP data sets. Note that values for the BE and NA data correspond to mean length of utterance (MLU) at first usage; whereas for the CDI data, values correspond to the children's age in months (parents reported child productions for months 16 to 31).

APPENDIX E: BASELINE MODELS (COVARIATES ONLY)

corpus + AoFP data	Effect (ΔR^2 in %)
BE corpus + NA-AoFP	43.2 %
NA corpus + BE-AoFP	37.2 %
joint BE-NA corpus + CDI-derived AoFP	24.2 %

Table 8. Amount of variance in AoFP (ΔR^2 in %) that can be explained by regression models which only include the covariates.

APPENDIX F: PROPORTION OF TARGET WORDS CONTAINED IN CHUNK SETS

BE corpus			NA corpus			BE + NA corpus		
freq.	pred.	short	freq.	pred.	short	freq.	pred.	short
19.1 %	21.0 %	29.6 %	24.6 %	25.1 %	36.8 %	74.4 %	72.0 %	85.7 %

Table 9. Proportion of target words contained in chunk sets consisting of the 10,000 most frequent, 10,000 most internally predictable, and 10,000 shortest MSUs. Chunk sets are taken from the BE corpus (paired with target words from the NA-AoFP data), the NA corpus (BE-AoFP), and the age-restricted joint corpus (CDI-derived AoFP).

APPENDIX G: AGE OF FIRST EXPOSURE FOR MULTI-SYLLABLE-UTTERANCES

It is possible that the results presented in analysis III are due to children being exposed to short MSUs before they are exposed to particularly frequent or internally predictable MSUs. Table 10 below contains the minim, maximum, and average age (in months) at which children were first exposed to the items in the chunk sets containing the 10,000 shortest, 10,000 most frequent, and 10,000 most internally predictable MSUs. The average age of first exposure for short MSUs is either similar to (BE + NA corpus) or larger than the age of first exposure for the other two MSU types (BE and NA corpus). Thus, the stronger effect for $\#MSU\text{-}S$ in analysis III is unlikely to be a result of earlier exposure to short MSUs.

measure	BE corpus			NA corpus			BE + NA corpus		
	freq.	pred.	short	freq.	pred.	short	freq.	pred.	short
min	17.7	17.7	17.7	3.0	3.0	3.0	3.0	3.0	3.0
max	61.1	86.1	86.8	114.8	228.0	228.0	30.0	30.0	30.0
mean	24.3	28.2	28.4	16.2	25.6	26.0	22.1	22.5	22.4

Table 10. Minimum, maximum, and average age (in months) at which children in the three corpora first heard the items contained in chunk sets covering the 10,000 most frequent, 10,000 most internally predictable, and 10,000 shortest MSUs. Note that the BE + NA corpus was restricted to only contain MSUs produced in the presence of children aged 30 months or less.

APPENDIX H: RESULTS WITHOUT COVARIATES

Figures 7, 8, and 9 are fashioned after figures 4, 5, and 6 from analysis III, except that we do not control for co-variates. In contrast to analysis III, we find that $MSU\text{-}F$ performs slightly better than $MSU\text{-}P$ when the co-variates are excluded (figure 9). Importantly, however, we replicate the key finding from analysis III: As long as N is not very small or very large, high $MSU\text{-}S$ counts are more strongly predictive of early AoFP than either high $MSU\text{-}F$ (figure 7) or high $MSU\text{-}P$ (figure 8) counts.

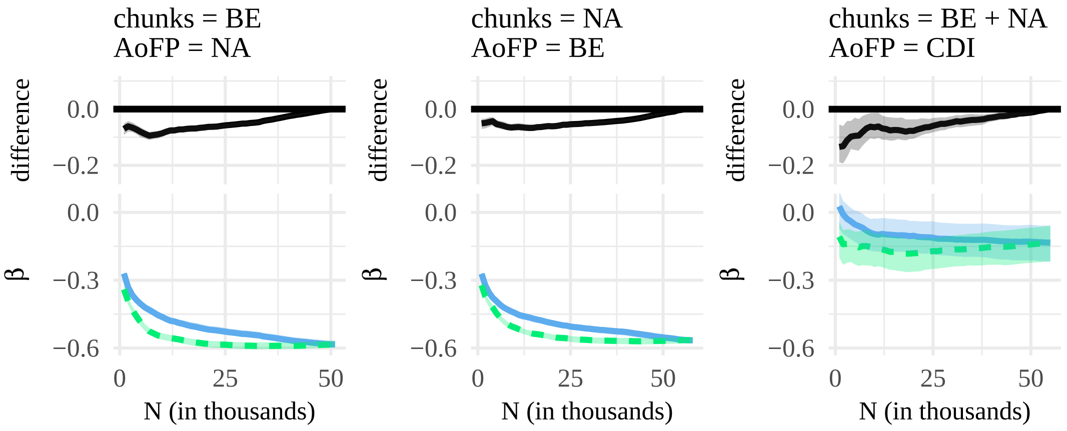

(7a) Bottom: Regression coefficients (β). Top: difference between coefficients.

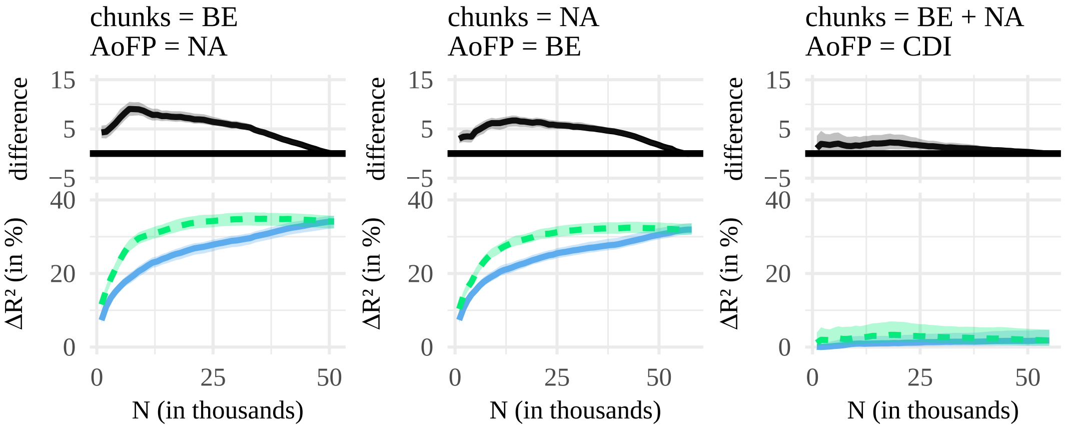

(7b) Bottom: amount of variance in AoFP (ΔR^2 in %) . Top: difference between R^2 values.

Figure 7. Comparison of $\#MSU\text{-}S$ (green line) and $\#MSU\text{-}F$ (blue line).

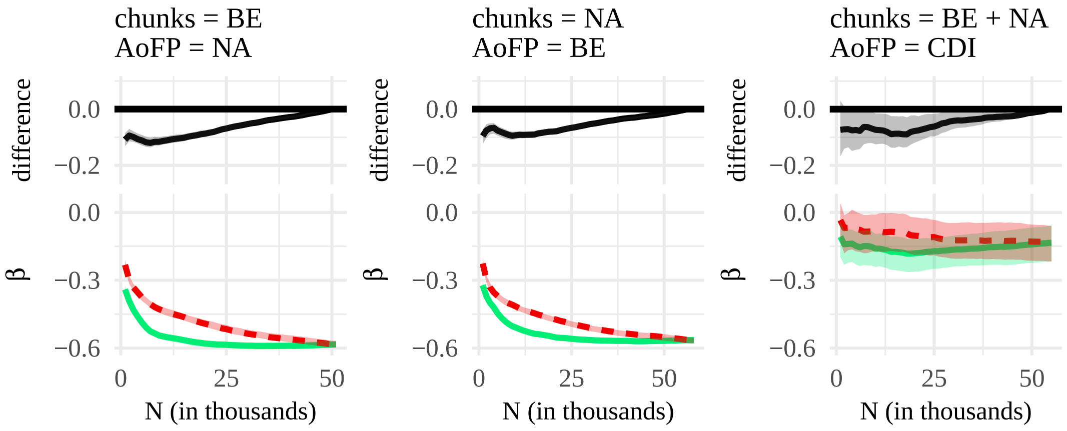

(8c) Bottom: Regression coefficients (β). Top: difference between coefficients.

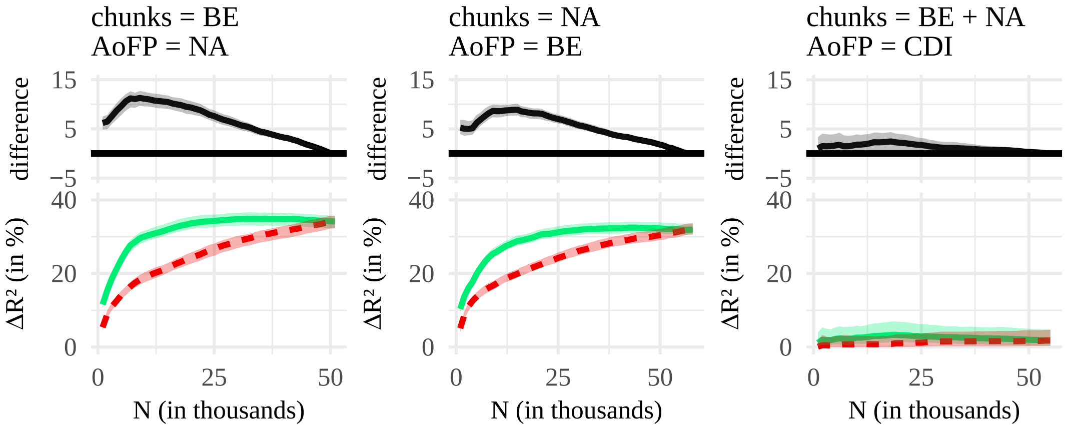

(8d) Bottom: amount of variance in AoFP (ΔR^2 in %) . Top: difference between R^2 values.

Figure 8. Comparison of $\#MSU\text{-}S$ (green line) and $\#MSU\text{-}P$ (red line).

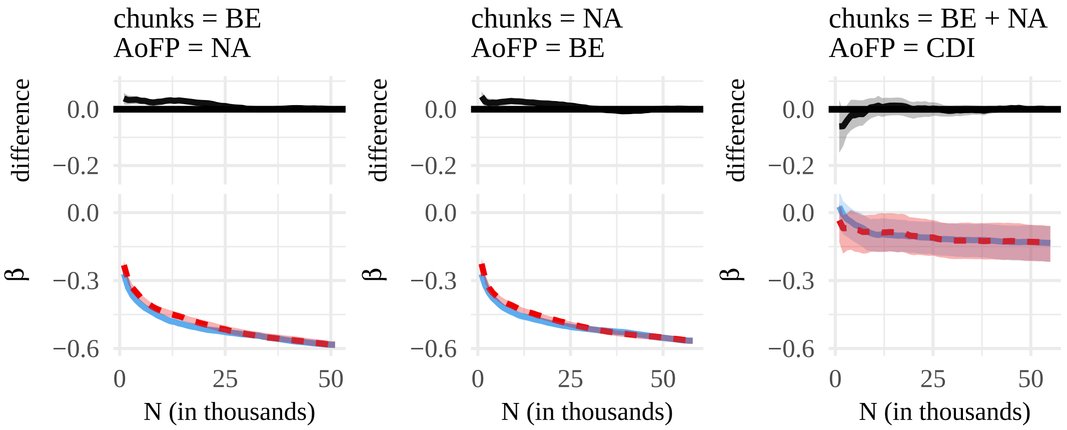

(9e) Bottom: Regression coefficients (β). Top: difference between coefficients.

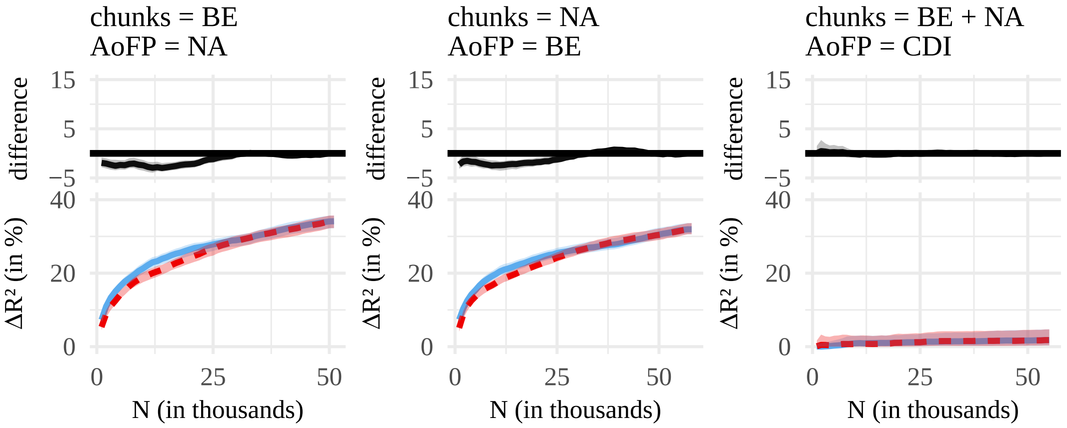

(9f) Bottom: amount of variance in AoFP (ΔR^2 in %) . Top: difference between R^2 values.

Figure 9. Comparison of $\#MSU$-P (red line) and $\#MSU$-F (blue line).

REFERENCES

Bates, E., Bretherton, I., and Snyder, L. (1991). *From first words to grammar: Individual differences and dissociable mechanisms* (Cambridge: Cambridge University Press)

Beals, D. E. (1993). Explanatory talk in low-income families' mealtime conversations. *Applied Psycholinguistics* 14, 489–513

Bliss, L. (1988). The development of modals. *Journal of Applied Developmental Psychology* 9, 253–261

Bloom, L. (1976). *One word at a time: The use of single word utterances before syntax* (Berlin: Walter de Gruyter)

Bloom, L., Hood, L., and Lightbown, P. (1974). Imitation in language development: If, when, and why. *Cognitive Psychology* 6, 380–420

Bohannon III, J. N. and Marquis, A. L. (1977). Children's control of adult speech. *Child Development* 80, 1002–1008

Braunwald, S. R. (1971). Mother-child communication: The function of maternal-language input. *Word* 27, 28–50

Brent, M. R. and Siskind, J. M. (2001). The role of exposure to isolated words in early vocabulary development. *Cognition* 81, B33–B44

Brown, R. (1973). *A first language: The early stages* (Cambridge, Massachusetts: Harvard University Press)

Brysbaert, M., Warriner, A. B., and Kuperman, V. (2014). Concreteness ratings for 40 thousand generally known english word lemmas. *Behavior Research Methods* 46, 904–911

Clark, E. V. (1978). Awareness of language: Some evidence from what children say and do. In *The child's conception of language*, eds. R. J. A. Sinclair and W. Levelt (Berlin: Springer Verlag). 17–43

Demetras, M. J., Post, K. N., and Snow, C. E. (1986). Feedback to first language learners: The role of repetitions and clarification questions. *Journal of Child Language* 13, 275–292

Demetras, M. J.-A. (1986). Working parents conversational responses to their two-year-old sons. *Working Paper. University of Arizona.*

Feldman, A. and Menn, L. (2003). Up close and personal: A case study of the development of three english fillers. *Journal of Child Language* 30, 735–768

Fletcher, P. and Garman, M. (1988). Normal language development and language impairment: Syntax and beyond. *Clinical Linguistics & Phonetics* 2, 97–113

Forrester, M. A. (2002). Appropriating cultural conceptions of childhood participation in conversation. *Childhood* 9, 255–276

Garvey, C. and Hogan, R. (1973). Social speech and social interaction: Egocentrism revisited. *Child Development* 44, 562–568

Hall, W. S., Nagy, W. E., and Linn, R. L. (1984). *Spoken words, effects of situation and social group on oral word usage and frequency* (Hillsdale, NJ: Lawrence Erlbaum)

Henry, A. (1995). *Belfast English and Standard English: Dialect variation and parameter setting* (New York: Oxford University Press)

Howe, C. (1981). *Acquiring language in a conversational context* (New York: Academic Press)

Kuczaj, S. A. (1977). The acquisition of regular and irregular past tense forms. *Journal of Verbal Learning and Verbal Behavior* 16, 589–600

Lieven, E., Salomo, D., and Tomasello, M. (2009). Two-year-old children's production of multiword utterances: A usage-based analysis. *Cognitive Linguistics* 20, 481–507

MacWhinney, B. (2000a). *The CHILDES project: The database* (Oxfordshire: Psychology Press)

MacWhinney, B. (2000b). The childes project: Tools for analyzing talk: Volume i: Transcription format and programs, volume ii: The database. *Computational Linguistics* 26, 657–657

MacWhinney, B. and Snow, C. (1990). The child language data exchange system: An update. *Journal of Child Language* 17, 457–472

Masur, E. F. and Gleason, J. B. (1980). Parent–child interaction and the acquisition of lexical information during play. *Developmental Psychology* 16, 404–409

McCune, L. (1995). A normative study of representational play in the transition to language. *Developmental Psychology* 31, 198

Morisset, C. E., Barnard, K. E., and Booth, C. L. (1995). Toddlers' language development: Sex differences within social risk. *Developmental Psychology* 31, 851

Ninio, A., Snow, C. E., Pan, B. A., and Rollins, P. R. (1994). Classifying communicative acts in children's interactions. *Journal of Communication Disorders* 27, 157–187

Ratner, N. B. (1986). Durational cues which mark clause boundaries in mother-child speech. *Journal of Phonetics* 14, 303–309

Rollins, P. (2003). Caregiver contingent comments and subsequent vocabulary comprehension. *Applied Psycholinguistics* 24, 221–234

Rowland, C. F. and Fletcher, S. L. (2006). The effect of sampling on estimates of lexical specificity and error rates. *Journal of Child Language* 33, 859–877

Sachs, J. (1983). Talking about the there and then: The emergence of displaced reference in parent-child discourse. *Children's Language* 4, 1–28

Sawyer, K. (1997). *Pretend play as improvisation* (Mahwah, New Jersey: Erlbaum)

Soderstrom, M., Blossom, M., Foygel, R., and Morgan, J. L. (2008). Acoustical cues and grammatical units in speech to two preverbal infants. *Journal of Child Language* 35, 869–902

Song, J. Y., Demuth, K., Evans, K., and Shattuck-Hufnagel, S. (2013). Durational cues to fricative codas in 2-year-olds' american english: Voicing and morphemic factors. *The Journal of the Acoustical Society of America* 133, 2931–2946

Suppes, P. (1974). The semantics of children's language. *American Psychologist* 29, 103–114

Theakston, A. L., Lieven, E. V., Pine, J. M., and Rowland, C. F. (2001). The role of performance limitations in the acquisition of verb-argument structure: An alternative account. *Journal of Child Language* 28, 127–152

Tommerdahl, J. and Kilpatrick, C. D. (2013). The reliability of morphological analyses in language samples. *Language Testing* 31, 3–18

Valian, V. (1991). Syntactic subjects in the early speech of american and italian children. *Cognition* 40, 21–81

Van Houten, L. J. (1986). The role of maternal input in the acquisition process: The communicative strategies of adolescent and older mothers with the language learning children. *Paper presented at the Boston University Conference on Language Development, Boston*

Warren-Leubecker, A. and Bohannon III, J. N. (1984). Intonation patterns in child-directed speech: Mother-father differences. *Child Development* 55, 1379–1385

Weist, R. M. and Zevenbergen, A. A. (2008). Autobiographical memory and past time reference. *Language Learning and Development* 4, 291–308

Wells, G. (1981). *Learning through interaction: The study of language development* (Cambridge: Cambridge University Press)
